# Fine-Tuning Translation Kinetics Selection as the Driving Force of Codon Usage Bias in the Hepatitis A Virus Capsid

**DOI:** 10.1371/journal.ppat.1000797

**Published:** 2010-03-05

**Authors:** Lluís Aragonès, Susana Guix, Enric Ribes, Albert Bosch, Rosa M. Pintó

**Affiliations:** 1 Enteric Virus Laboratory, Department of Microbiology and Institute of Nutrition and Food Safety, University of Barcelona, Barcelona, Spain; 2 Enteric Virus Laboratory, Department of Cell Biology, University of Barcelona, Barcelona, Spain; University of California San Francisco, United States of America

## Abstract

Hepatitis A virus (HAV), the prototype of genus *Hepatovirus*, has several unique biological characteristics that distinguish it from other members of the *Picornaviridae* family. Among these, the need for an intact eIF4G factor for the initiation of translation results in an inability to shut down host protein synthesis by a mechanism similar to that of other picornaviruses. Consequently, HAV must inefficiently compete for the cellular translational machinery and this may explain its poor growth in cell culture. In this context of virus/cell competition, HAV has strategically adopted a naturally highly deoptimized codon usage with respect to that of its cellular host. With the aim to optimize its codon usage the virus was adapted to propagate in cells with impaired protein synthesis, in order to make tRNA pools more available for the virus. A significant loss of fitness was the immediate response to the adaptation process that was, however, later on recovered and more associated to a re-deoptimization rather than to an optimization of the codon usage specifically in the capsid coding region. These results exclude translation selection and instead suggest fine-tuning translation kinetics selection as the underlying mechanism of the codon usage bias in this specific genome region. Additionally, the results provide clear evidence of the Red Queen dynamics of evolution since the virus has very much evolved to re-adapt its codon usage to the environmental cellular changing conditions in order to recover the original fitness.

## Author Summary

Each organism has a specific codon usage signature. Translational selection i.e., selection for the codon adaptation to the tRNA pools, is one of the driving forces of codon bias. In the virus world, this implies an adjustment of the virus codon usage to that of the host cell. Hepatitis A virus appears as an exception to the rule, with a highly deoptimized codon usage, suggesting that translational selection is not the underlying mechanism of its codon bias. However, since the virus lacks a mechanism of cellular protein synthesis inhibition, the deoptimized codon usage may be envisaged as a hawk (cell) and dove (hepatitis A virus) competition strategy for tRNAs and translational selection as well. To confirm this possibility, we artificially induced cell protein synthesis shut-off, thus increasing the tRNA pool availability for the virus, and we took advantage of the quasispecies dynamics to elucidate changes in its codon usage. Virus adaptation to the drug results in a re-deoptimization of codon usage in the capsid region, suggesting a requirement of a slow translation rate, i.e., a translation kinetic selection, instead of a translational selection associated with an optimization of the codon usage. Translation kinetics control is based on the right combination of codons (common and rare) that allows a regulated ribosome traffic rate ensuring the proper protein folding. Capsid folding is critical for a virus transmitted through the fecal-oral route with long extracorporeal periods.

## Introduction

Non-random usage of synonymous codons is a widespread phenomenon observed in genomes from many species in all domains of life and it has been proposed that each genome has a specific codon usage signature that reflects particular evolutionary forces acting within that genome [1],[2]. Such codon usage biases may result from mutational biases, from natural selection acting on silent changes in the genomes or both. Selection on codon usage due to codon adaptation to the tRNA pool was clearly demonstrated in *Escherichia coli* [3]. Later on, this premise was also confirmed in eukaryotic organisms including vertebrates [4]–[8], supporting the hypothesis of translational selection as a driving evolutionary force. However, this hypothesis is not extensive to all organisms and in humans there is no clear evidence of translation selection as the single driving force of codon bias [9]. In this latter case many different reasons have been identified to explain codon bias: isochoric structure of GC content [10], mRNA secondary structure selection [11], exonic splicing enhancer selection [12],[13], and last but not least translation selection [14],[15]. Additionally, there is also evidence of pressure on codon usage for the control of translation kinetics rather than for the efficiency and accuracy of translation [16],[17]. Translation kinetics control is exerted through the use of common and rare codons, which affect the rate of ribosome traffic on the mRNA due to the longer time required for incorporating those tRNAs pairing with rare codons into the ribosome A-site. The right combination of codons allows a regulated ribosome traffic rate that temporally separates protein folding events, ensuring “beneficial” and avoiding “unwanted” interactions within the growing peptide [18]. This kind of selection, known as fine-tuning translation selection, differs from translation selection in that preferred codons are not always advantageous if the optimal folding requires a slow translation.

A statistical model for translational selection measure was developed based on fully sequenced genomes from archaea, bacteria and eukarya [19]. This model shows that small genome size allows higher action of translation selection, whilst lack of tRNA gene redundancy accounts for the absence of translationally selected codons. Bearing in mind this model, it seems reasonable that translation selection might play a key role on eukaryotic viruses, which present tiny genomes and have huge numbers of tRNA genes available from their hosts. Certainly codon bias has been observed in several viruses, however, its driving force has only been studied in particular cases, such as Epstein-Barr Virus [20] and papillomavirus [21] among DNA viruses, and poliovirus (PV) [22] and hepatitis A virus (HAV) [23] among RNA viruses. While translation selection seems to be the underlying mechanism of the codon bias of those genes expressed during the productive phase in the DNA viruses, the mechanism in the RNA viruses is more variable. The codon usage bias in the capsid of PV, which presents a highly optimized codon usage with that of its host, has been proposed to be mostly the result of an additional step on translation selection, i.e., the effect of certain codon-pair combinations on the rate of translation [24] rather than only the tRNA availability. This may be due to the fact that translational step times are influenced by the compatibilities of adjacent tRNA isoacceptor molecules on the surface of a translating ribosome [25]. Alternatively, it has also been proposed the GC dinucleotide content as the cause of codon bias in the capsid of PV [22].

However, HAV is clearly the exception to the rule. HAV presents a highly biased codon usage and mostly opposed to that of the host cell [23]. Despite lacking mechanisms of inducing cellular shutoff [26],[27] and having a very inefficient IRES [28], HAV is able to synthesize its proteins by adapting their codon usage to those less commonly used cellular tRNAs, resulting in a low replication rate [29]. With that naturally deoptimized codon usage the role of translation selection in shaping the HAV codon bias does not seem very obvious.

HAV is the type species of the genus *Hepatovirus* within the family *Picornaviridae*, existing as a single serotype. The occurrence of highly conserved clusters of rare codons in the HAV capsid-coding region has been related to the low antigenic variability [30], since mutations in these clusters are negatively selected even in the presence of immune pressure. Thus, a certain beneficial role of these rare codons is envisaged. Altogether, it can be concluded that codon usage plays a key role in HAV replication and evolution.

To elucidate the underlying mechanisms of the naturally deoptimized codon usage of HAV, a system to cultivate the virus in a cellular environment with a modified tRNA pool was developed. This was achieved using actinomycin D (AMD), which specifically inhibits the DNA-dependent RNA polymerases with no effect on the RNA-dependent RNA polymerases [31]. In these conditions of cellular transcription inhibition and hence cellular protein synthesis shut-off, the tRNA pool available for the virus is expected to increase and consequently the virus codon usage may readapt to the new conditions. HAV, being an RNA virus, replicates as a complex and dynamic mutant spectrum or swarm of non identical but very closely related individuals, called viral quasispecies [32],[33]. The population landscape of these molecular spectra allows a more broad analysis of all ongoing mutations necessary for the study of codon usage adaptation. The response regarding the viral replicative fitness and codon usage assessed during the adaptation to AMD exclude translation selection and instead suggest fine-tuning translation kinetics selection as the underlying mechanism of the codon usage bias in the capsid coding region.

## Results

### Viral replication in conditions of cellular shut-off: fitness loss and fitness recovery

**Figure 1:**
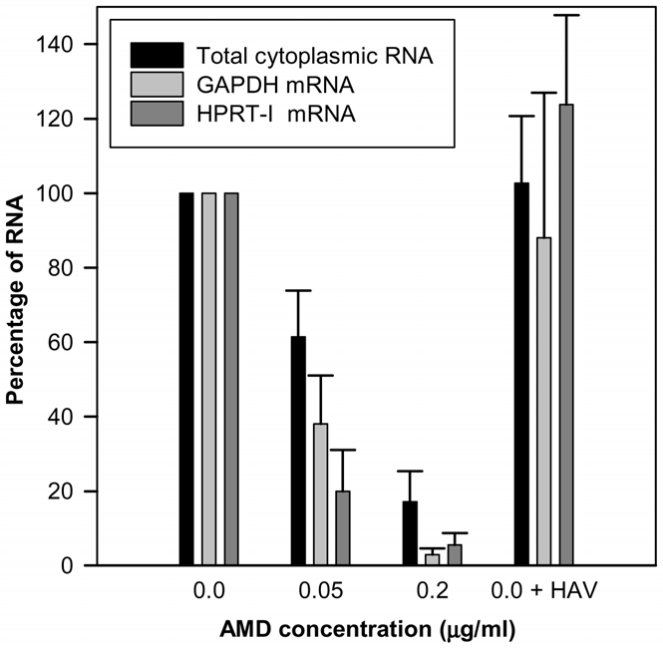
Abundance (percent) of total cytoplasmic RNA and mRNA from two house-keeping genes (GAPDH and HPRT-I) in uninfected FRhK-4 cell monolayers growing in the absence of AMD, in the presence of 0.05 µg/ml and 0.2 µg/ml of AMD, and in HAV-infected cells in the absence of the drug.

Actinomycin D (AMD) treatment induced a clear dose-dependent inhibition of gene expression of FRhk-4 cells with a total cytoplasmic RNA reduction of around 40% and over 80% at concentrations of 0.05 µg/ml and 0.2 µg/ml, respectively (Fig. 1). Additionally, the expression of the housekeeping genes HPRT-I and GAPDH was also significantly reduced by 62% and 80%, respectively, at 0.05 µg/ml of AMD and 97% and 94%, respectively, at 0.2 µg/ml of AMD. In contrast, neither the cellular RNA abundance nor the housekeeping gene expression was significantly affected as a consequence of HAV replication. The AMD-induced cellular shut-off resulted in a cell viability of 75% and 0.25% with 0.05 µg/ml and 0.2 µg/ml, respectively, at 7 days post-treatment; of around 100% and 10%, respectively, at 4 days post-treatment, and of 100% and 60%, respectively, at 2 days post-treatment.

**Figure 2:**
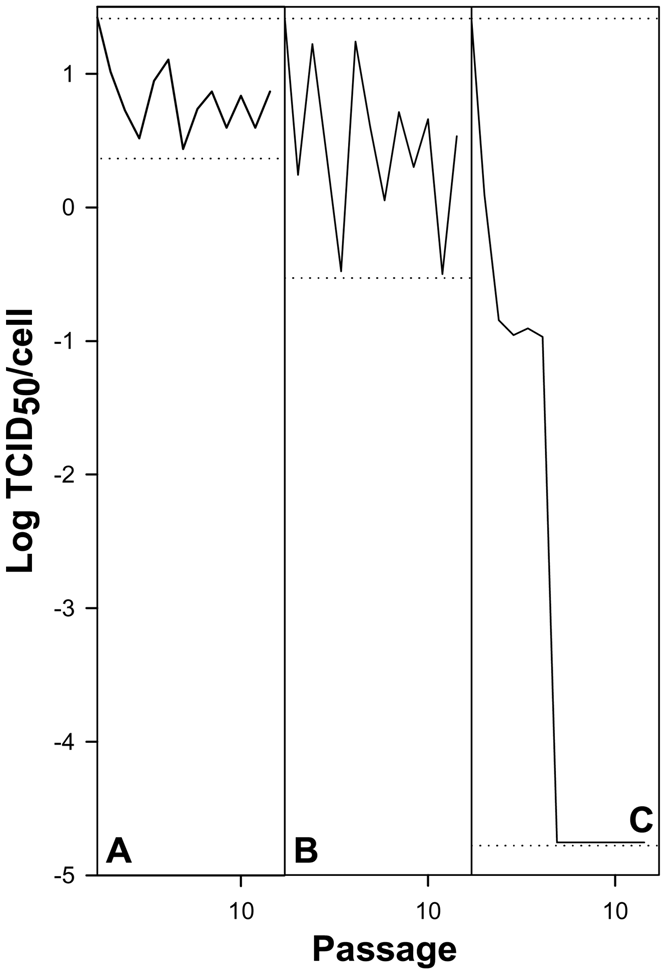
HAV yield as a measure of viral fitness in the absence and presence of AMD. A. 0.0 µg/ml of AMD. B. 0.05 µg/ml of AMD. C. 0.2 µg/ml of AMD. Dotted lines depict maximum and minimum virus yields per cell.

**Figure 3:**
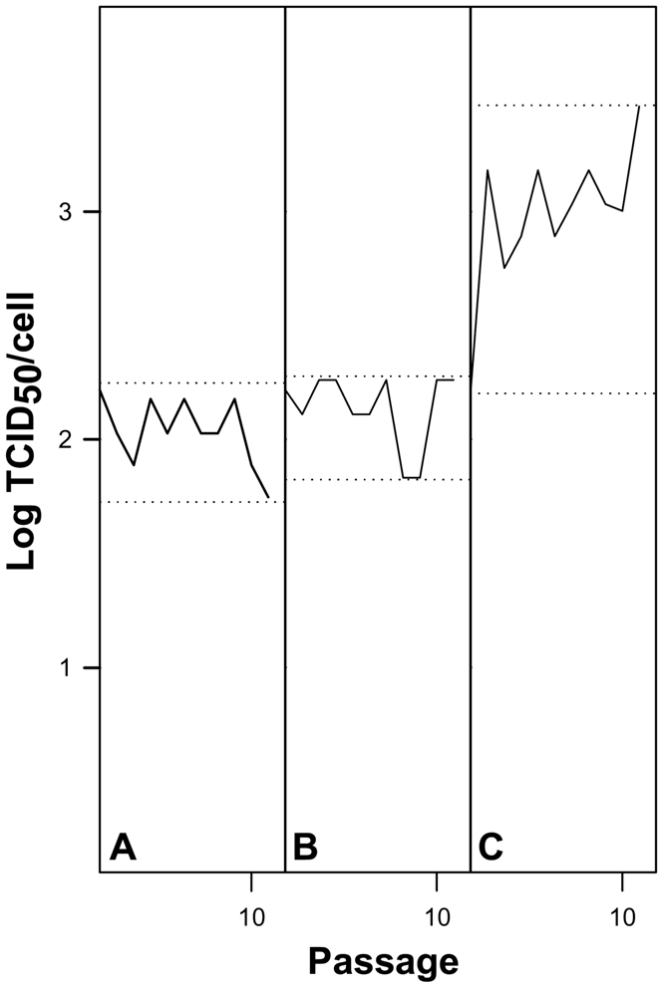
PV yield as a measure of viral fitness in the absence and presence of AMD. A. 0.0 µg/ml of AMD. B. 0.05 µg/ml of AMD. C. 0.2 µg/ml of AMD. Dotted lines depict maximum and minimum virus yields per cell.

The effect of AMD-induced cellular shut-off on viral fitness was analyzed in an attempt to reproduce what occurs in other picornaviruses such as poliovirus. In the presence of 0.05 µg/ml of AMD, and during the early passages, HAV showed a loss in fitness (figured as virus production per cell), and at 0.2 µg/ml of AMD concentration the virus population was completely extinct (Fig. 2). In contrast PV, which induces cellular shut-off by itself and that has an optimized codon usage, readily showed a fitness gain in the presence of 0.2 µg/ml of AMD (Fig. 3).

**Figure 4:**
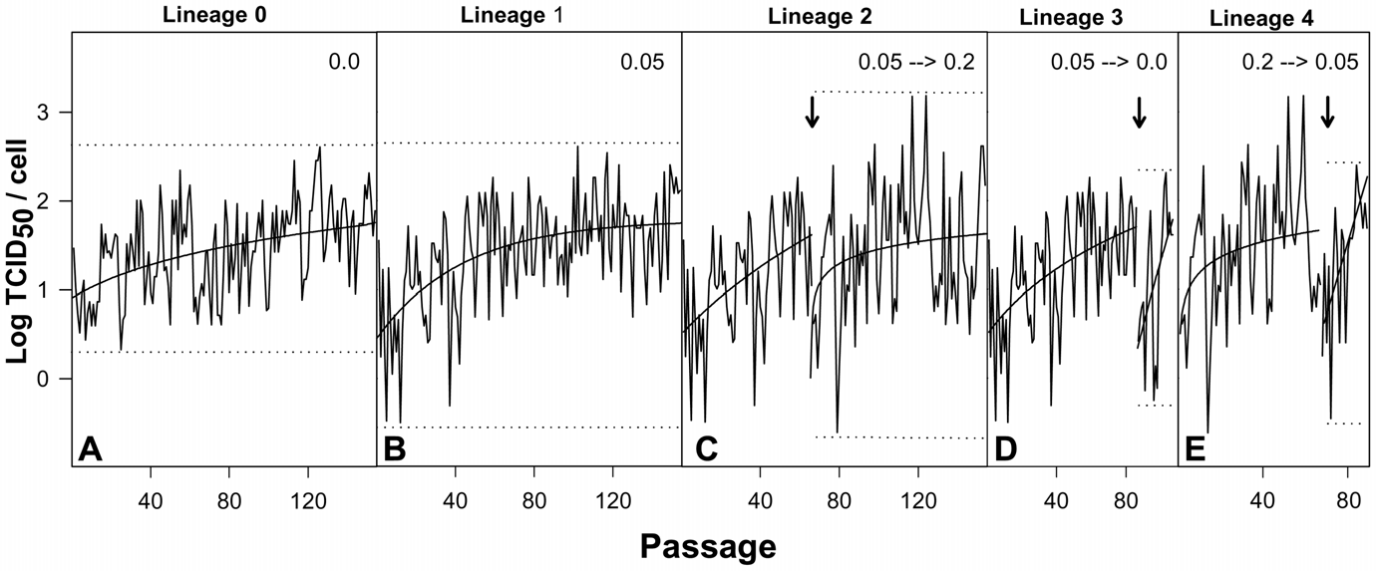
HAV adaptation to different AMD conditions. A. As a control, the virus population was grown in the absence of the drug (0.0 µg/ml of AMD; lineage 0). B. Adaptation of the virus population to 0.05 µg/ml of AMD (0.05 µg/ml of AMD; lineage 1). C. At passage 65, lineage 1 population was transferred to 0.2 µg/ml of AMD (0.05 µg/ml of AMD →0.2 µg/ml AMD; lineage 2). D. At passage 85, lineage 1 population was transferred back to 0.0 µg/ml of AMD (0.05 µg/ml of AMD →0.0 µg/ml of AMD; lineage 3). E. At passage 65, lineage 2 population was transferred back to 0.05 µg/ml of AMD (0.2 µg/ml of AMD →0.05 µg/ml of AMD; lineage 4). Dotted lines depict maximum and minimum virus yields per cell. Arrows depict changes in AMD conditions.

To find out whether HAV could recover its fitness through a change of its codon usage, further passages in the presence of the drug were performed (Fig. 4). Replication in the presence of 0.05 µg/ml of AMD (lineage 1) was examined for over 150 passages (Fig. 4B) using as baseline control the viral replication in the absence of AMD (lineage 0) (Fig. 4A). As aforementioned, after few passages in the presence of the drug, viral production was severely decreased, with peaks of less than 1 TCID_50_ per cell (Fig. 4B). However, thereafter the viral population progressively adapted to AMD, giving rise again to viral progenies quantitatively equivalent to those of the population growing in absence of the drug. Additionally, after 65 passages the AMD-pre-adapted population was submitted to an increased concentration of the drug (0.2 µg/ml) (lineage 2), and again a decrease followed by a clear recovery in the viral production was observed (Fig. 4C). To further test the fitness of the populations adapted to the different AMD concentrations, viral populations were again submitted to the original conditions. For instance, once adapted to 0.05 µg/ml (lineage 1), the virus population was brought back to grow in the absence of the drug (lineage 3) (Fig. 4D). The immediate response was again an abrupt decrease in the viral progeny that, however, was very rapidly recovered. Similarly, when the 0.2 µg/ml AMD-adapted population (lineage 2) was brought back to the original 0.05 µg/ml concentration (lineage 4), the same pattern of behavior of an initial loss in fitness followed by a fitness recovery was observed (Fig. 4E). Nevertheless, the analysis of the viral production of the first 20 passages of the different adaptive processes showed that the kinetics of adaptation was faster during the re-adaptation to the original conditions than during the first adaptation to the different AMD concentrations, with significantly (p<0.05) different slopes of the regression lines (Fig. S1).

### Codon usage in depleted *vs* abundant tRNA pools: codon usage adapts to tRNA concentration

**Figure 5:**
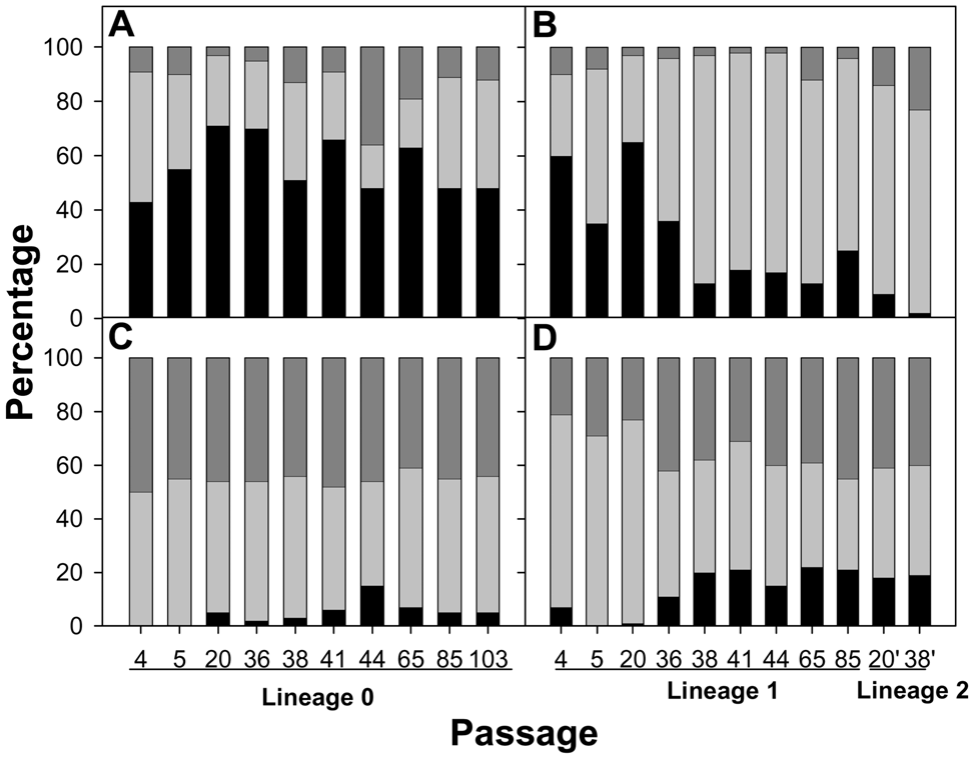
Relative proportion (percentage) of the new generated codons detected during the process of HAV adaptation to AMD. These codons were sorted as being similarly frequent (black fill), less frequent (gray fill) or more frequent (blue fill) than the original ones with respect to the cell host codon usage. A. Codon usage variation in the capsid region in the absence of AMD (lineage 0). B. Codon usage variation in the capsid region in the presence of 0.05 µg/ml of AMD (lineage 1) from passage 4 to passage 85, and in the presence of 0.2 µg/ml of AMD (lineage 2) from passage 20 (P20') to passage 38 (P38'). C. Codon usage variation in the polymerase region in the absence of AMD (lineage 0). D. Codon usage variation in the polymerase region in the presence of 0.05 µg/ml of AMD (lineage 1) from passage 4 to passage 85, and in the presence of 0.2 µg/ml of AMD (lineage 2) from passage 20 (P20') to passage 38 (P38').

The Relative Codon Deoptimization Index (RCDI) [34] measures the adaptation of a virus codon usage to that of its host. An RCDI value of 1 indicates the virus follows the cell host codon usage, while the higher the value the higher the deviation from the host. Thus, a value of around 1.70 denotes that HAV posses a highly deoptimized codon usage compared to other picornaviruses whose RCDI range from 1.14 to 1.39 (Table S1). Since HAV is not able to induce cellular shut-off [26],[27] (Fig. 1), the optimization of the viral codon usage to that of the host cell could lead to an unfair competition for tRNA resources. In contrast replication in the presence of AMD, which clearly inhibits cellular expression, may represent an environment with increased available tRNA pools. Hence, an analysis to test the adaptation to tRNA pools was performed by studying the mutant spectrum of each adapting lineage at different passages and determining the codon usage in each of these spectra. Particularly passages P4, P5, P20, P36, P38, P41, P44, P65 and P85 of lineage 1 and P20 and P38 of lineage 2 (P20' and P38' in Fig. 5) were analyzed. Additionally, the mutant spectrum of lineage 3 was also analyzed at P21. In each case the quasispecies distribution of lineage 0 was used as baseline control of the molecular evolution. Three genomic regions were analyzed: two fragments from the structural polyprotein coding region (a VP3 fragment and a VP1 fragment) and a fragment from the polymerase coding region.

Any mutation induces a codon change and the newly generated codons were classified as being similarly frequent (within a 10% range), less frequent (below 10%) or more frequent (above 10%) than the original ones with respect to the cell host codon usage, as an indication of the adaptation to the cellular tRNA pool. A different pattern of molecular evolution was observed depending on the genomic region analyzed. The structural polyprotein coding regions of the AMD-adapting populations showed a tendency to progressively accumulate mutations that induced the use of codons less common than the original ones (Fig. 5B), with an average of 75% of mutations in this direction in both the last passages of lineage 1 and all passages of lineage 2. On the contrary, the new generated codons in lineage 0 were mostly similar to the original ones (Fig. 5A). Particularly, 54% of the mutations gave rise to codons of the same frequency, 31% to less frequent codons and 15% to more common codons. Quite the opposite, in the polymerase region a complete dominance of mutations inducing changes in the level of frequency (almost equally in both directions less and more frequent) of the new codons was observed in all lineages (Fig. 5C and 5D).

Assuming a parallel behavior between the particular capsid coding regions analyzed and the complete capsid coding region, and applying the Poisson distribution model, it may be postulated that while at P5, during the adaptation to 0.05 µg/ml of AMD, around 50% of the genomes would present only 1 mutation inducing the generation of a less common codon, from P38 and further 50% of the genomes would harbor at least 5 of such mutations. In other words, the percentage of genomes with zero mutations of this kind would progress from 20% at P5 to 0.20% at P85.

**Figure 6:**
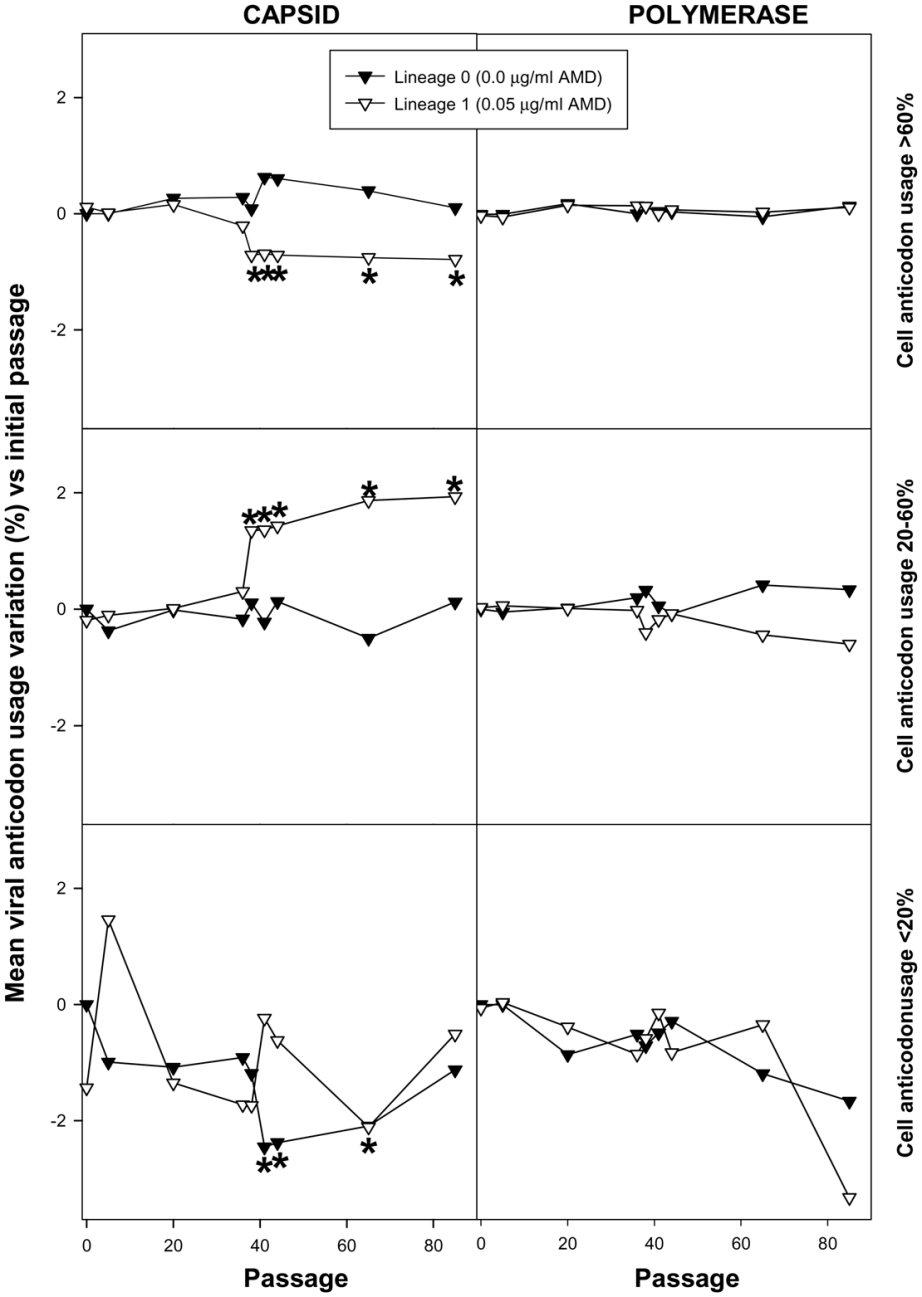
HAV anticodon usage variation in the capsid and polymerase regions during the adaptation to 0.05 µg/ml of AMD. Anticodons were sorted in three groups based on the cellular anticodon usage: those used by the cell at a proportion above 60%, those used at a proportion between 20 to 60% and those used at a proportion below 20%. The mean percent viral anticodon usage variation was calculated in the mutant spectra with respect to the initial passage for each group of anticodons at each passage. Asterisks depict significant differences (p<0.05) between the codon usage variation in a given passage with respect to the initial passage.

**Figure 7:**
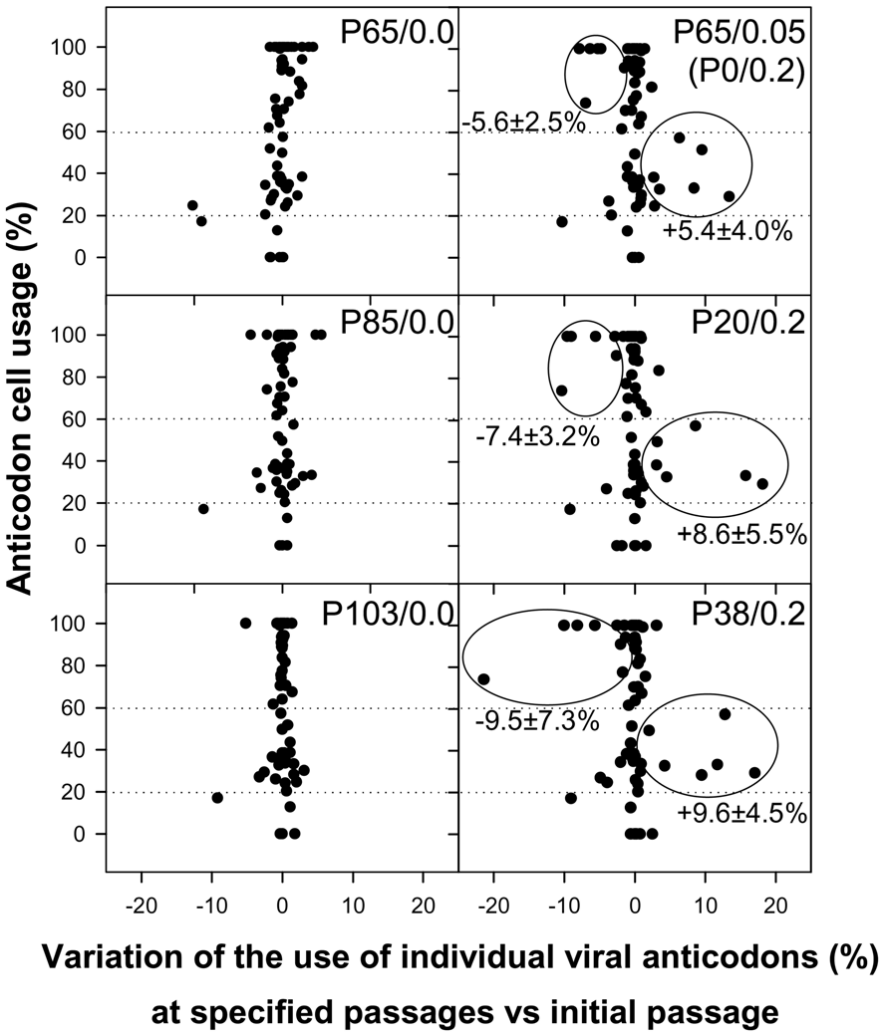
HAV anticodon usage variation in the capsid coding region during the adaptation to 0.2 µg/ml of AMD. Passage 65 of the population adapted to 0.05 µg/ml of AMD was thereafter passaged in the presence of 0.2 µg/ml of the drug (lineage 2; right column). Left column depicts the behavior of the population in the absence of AMD as baseline control. The percentage of variation of each individual viral anticodon in the mutant spectra with respect to the cellular usage is shown. Mean and standard deviation of the variation of use of those anticodons with the highest level of variation (encircled) is shown.

Since competition is established for the tRNA resources, a refined analysis involving the study of anticodon usage was performed by inferring theoretical anticodon usage tables from the actual codon usage tables which were built from the analysis of 50 molecular clones representative of a mutant spectrum (Table S4, A and S5, A), but introducing the codon-anticodon multiple pairing effects corrected by the codon-anticodon coupling efficiency [19]. Two codon usage tables were made for each viral population at each analyzed passage, one using the mutant spectra in the VP1- and VP3-region analyzed (Table S4, A) and a second one using the mutant spectrum of the 3D-region analyzed (Table S5, A). Likewise, two anticodon usage tables were built: one for the capsid region (Table S4, B) and another for the polymerase region (Table S5, B). The relative anticodon usage for each amino acid family was also figured (Tables S4, C and S5, C). Additionally, an anticodon usage table for the host cell was also built and a relative anticodon usage determined by arbitrarily giving to the most abundant anticodon in each amino acid family a value of 100% and the remaining anticodons were percentualy referred to this most abundant one. Anticodons were then sorted in three groups following this cellular anticodon usage table: anticodons used by the cell at a proportion below 20% (<20%), anticodons used by the cell at a proportion between 20% and 60% (20%–60%) and anticodons used by the cell at a proportion above 60% (>60%). The viral relative anticodon usage variation of each population in each passage, with respect to the initial passage in the absence of the drug, was calculated (Table S4, D and Table S5, D). Increases or decreases (in percentage) of use of the anticodons belonging to the above mentioned groups were analyzed and the mean variation figured (Fig. 6). Lineage 0 showed no major statistical (p<0.05) variation of the anticodon use, with the exception of the anticodon group <20% in the capsid region at P41, P44 and P65 which decreased (Fig. 6). In contrast, lineage 1 showed a significant (p<0.05) and consistent tendency, in the capsid region, to decrease the anticodon group >60% and increase the anticodon group 20%–60% (Fig. 6). This tendency was further confirmed with lineage 2 at P20 and P38 (Fig. 7). Such a tendency was not observed in the polymerase region (Fig. 6). Five anticodons (Ile: uag, uai; Thr: ugg; Val: cau, cac) among the >60% group were responsible for the decrease of the whole group, being their decrease at P65 of lineage 1 of −5.6%±2.5%. Further on this group decreased from P20 to P38 of lineage 2 from −7.4%±3.2% to −9.5%±7.3% (Fig. 7). All these variations were significantly (p<0.05) different from the values shown by the same anticodons in lineage 0. On the other sense, the variation of the six anticodons (Arg: ucc; Ile: uaa, uau; Val: cag, cai, caa) of the 20%–60% group responsible of the increase of the whole group was also significantly (p<0.05) different from that of lineage 0 and of 5.4%±4.0% (P65 of lineage 1), 8.6%±5.5% (P20 of lineage 2) and 9.6%±4.5% (P38 of lineage 2) (Fig. 7). Interestingly, when lineage 1 was returned to the original growing conditions of absence of AMD (lineage 3) for 21 passages (P21R), the anticodon groups >60% and 20%–60% increased and decreased, respectively. However, although the five specific anticodons of the >60% group and the six specific anticodons of the 20%–60% group increased from −6.3%±3.5% to −1.1%±4.8% and decreased from 6.9%±3.9% to 3.5%±2.9%, respectively, these variations were not statistically significant. However, six additional anticodons belonging to the >60% group (Ala:cgg, cgi; Glu:cuc; Gly:ccc; His:gug; Pro:ggu) did significantly (p<0.05) increase an average of 7.8%±6.5% in comparison with the original lineage 1 (P0R) and four more from the 20%–60% group (Ala:cgc; Gly:cca; His:guc; Ser:ucg) significantly (p<0.05) decreased an average of −6.30%±1.7% (Fig. S2).

To confirm that the observations made with the molecular quasispecies analysis of the three specific regions may be inferred to the whole genome, consensus sequences at P127 of lineages 0 (absence of AMD) and 1 (presence of 0.05 µg/ml of AMD), and at P62 of lineage 2 (presence of 0.2 µg/ml of AMD) were obtained. Nine mutations characterized lineage 1. Three of them were located in the capsid region (µ = 1.3×10^−3^) and 6 at the non-structural proteins region (NSP) (µ = 1.4×10^−3^). Lineage 2 showed 16 mutations, one at the 5′ non coding region (µ = 1.4×10^−3^), 8 in the capsid region (µ = 3.4×10^−3^) and 7 at the NSP region (µ = 1.6×10^−3^). Most of the mutations occurring in the capsid region (63%) induced a change to a less frequent codon, as also occurred in the mutant spectra (75%), while at the NSP region they mainly induced the change to a more frequent or similar one (57% and 29%, respectively). Furthermore, the anticodon analysis was also performed and it showed the same trend. A decrease of the anticodon group of >60% was observed and the average variation of the five main anticodons (Ile: uag; Leu: gau; Phe: aag; Val: cau, cac) responsible for the change of the whole group in lineage 1 was −2.08% and the variation of the eight main anticodons (Ile: uag; Leu: gau; Phe: aag; Pro: ggg, ggi; Tyr: aug; Val: cau, cac) responsible for the change in lineage 2 was −3.03%. Also an increase in the 20%–60% group was detected, with variations of the six main responsible anticodons (Ile: uaa, uau; Leu: aac; Phe: aaa; Val: cag, cai) in lineage 1 of 4.92% and of the ten main responsible anticodons (Ile: uaa, uau; Leu: aac; Phe: aaa; Ser: aga, uca; Tyr: aua; Val: cag, cai, caa) in lineage 2 of 4.25%. Although the population landscapes obtained with consensus sequences are less representative than those obtained with the molecular spectra, this is in some way compensated by the analysis of a wider length and the results observed support the conclusions from the molecular quasispecies analysis.

Moreover, during the process of adaptation to AMD, the RCDI of the capsid coding region significantly (p<0.05) increased (Fig. S3 and Table S1), indicating a re-deoptimization of the virus codon usage under the new growing conditions.

### Codon usage determines viral fitness in tRNA changing conditions

**Figure 8:**
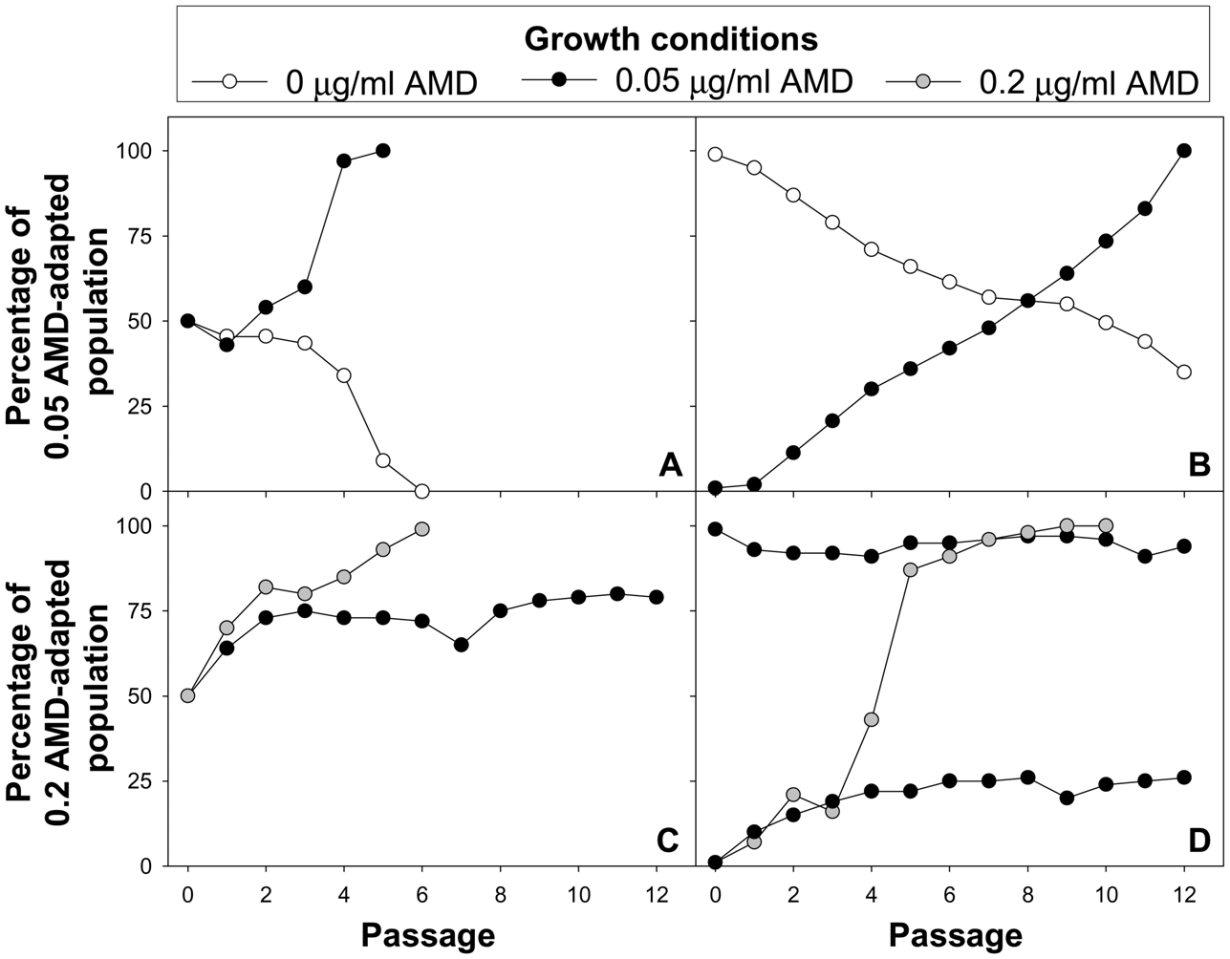
Growth competition experiments. A and B. Populations adapted to grow in the absence of AMD (lineage 0) and in the presence of 0.05 µg/ml of AMD (lineage 1) were mixed at different ratios and grown in the absence of AMD or with 0.05 µg/ml of AMD. Initial population mixture ratios were 1∶1 (lineage 0: lineage 1) in A, and 100∶1 (lineage 0: lineage 1) and 1∶100 (lineage 0: lineage 1) in B. C and D. Populations adapted to grow with 0.05 µg/ml (lineage 1) and 0.2 µg/ml of AMD (lineage 2) were mixed at different ratios and grown in the presence of 0.05 µg/ml of AMD or with 0.2 µg/ml of AMD. Initial population mixture ratios were 1∶1 (lineage 1: lineage 2) in C, and 100∶1 (lineage 1: lineage 2) and 1∶100 (lineage 1: lineage 2) in D.

To investigate whether codon usage plays a significant role on viral fitness in conditions of depleted and abundant tRNA pools, competition experiments between viral populations with codon usages adapted to replicate in the absence or presence of AMD were carried out. With this aim mixed populations at different quantitative ratios were grown under different conditions (Fig. 8). These experiments clearly demonstrated that lineage 1 (adapted to grow in 0.05 µg/ml of AMD) was the fittest in the presence of 0.05 µg/ml of AMD and as early as after 4 passages completely out-competed lineage 0 (adapted to grow in the absence of AMD) when mixed at equal concentrations (Fig. 8A). In the most quantitatively unfavorable condition, 12 passages were required to out-compete lineage 0 (Fig. 8B). On the contrary, this latter population was the fittest in the absence of the drug and clearly out-competed the drug-adapted population after 6 and 12 passages, when mixed at equal concentrations (Fig. 8A) and in quantitatively unfavorable condition (Fig. 8B), respectively.

In the presence of 0.2 µg/ml of AMD, lineage 2 (adapted to grow in 0.2 µg/ml of AMD) clearly showed a better fitness and rapidly out-competed lineage 1 (Fig. 8C) even in the most unfavorable condition that required only 9 passages (Fig. 8D). However, in the presence of 0.05 µg/ml of AMD, lineage 1 was never able to out-compete lineage 2 and this latter lineage, although showing a relative better fitness in this condition, was unable to totally out-compete lineage 1 when mixed at equal or unfavorable ratio (Fig. 8C and 8D). This relative better fitness is also evidenced by the incapacity of lineage 1 to affect lineage 2 in 0.05 µg/ml of AMD when the starting concentration of lineage 2 was highly favorable (Fig. 8D).

### HAV capsid folding is independent on the activity of the heat-shock protein 90 (Hsp90) chaperone

**Figure 9:**
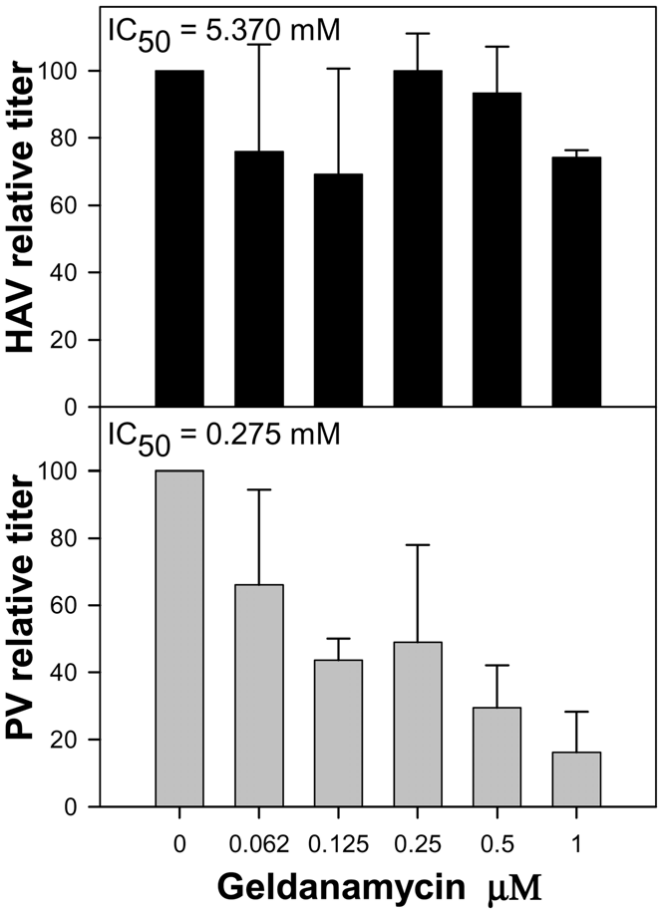
Relative infectious virus production per cell of HAV and PV in the presence of increasing concentrations of geldanamycin, a heat-shock protein 90 (Hsp90) inhibitor. Viral production at each geldanamycin concentration is expressed as a percentage of viral production in the absence of the drug.

The effect of the Hsp90 chaperone inhibitor geldanamycin on HAV production was investigated. HAV titers were not affected by the presence of increasing concentrations of the drug ranging from 0 to 1 µM. The estimated geldanamycin concentration inducing a 50% virus titer reduction (IC_50_) was 5.370 µM (Fig. 9). In contrast, PV titers were severely affected by the presence of the drug, with an estimated IC_50_ of 0.275 µM. Since in the particular case of PV, the decrease in titer is thought to be the result of an impairment of capsid folding, which is dependent on the activity of the Hsp90 [35], it can be assumed that HAV capsid folding is not dependent on the activity of this specific chaperone and that it should depend on other factors as might be the codon usage.

## Discussion

Translation selection drives the optimal co-adaptation of the codon usage and tRNA concentration, i.e. the most abundant codons pair with the most abundant tRNAs, in order to get a quantitatively highly efficient and accurate rate of translation [18]. In contrast, fine-tuning translation kinetics selection also presses for a co-adaptation between codon usage and tRNA concentration but in a different sense, i.e. the use of many different rare codons pairing with non-abundant tRNAs, in order to get a locally slow ribosome traffic rate to allow the proper protein folding [18]. The codon usage of HAV is indeed highly biased and highly deoptimized [23] with the highest RCDI value among picornaviruses, and thus translation selection does not seem to be the evolutionary driving force of its codon bias. In such a situation the viral translation rate is expected to be very slow, in agreement with a highly inefficient IRES [28], since the rate of translation is proportional to the concentration of charged tRNAs. We attempted to adapt HAV to grow in an environment with a higher tRNA availability through the specific inhibition of the cellular protein synthesis with AMD, and to study the codon usage re-adaptation, if any, to these new conditions. Although our initial hypothesis was that an environment of increased tRNA availability would result in an improvement of HAV viral translation rate, what we found during the first passages of the virus in the presence of AMD was a significant decrease of the infectious viral production per cell (Fig. 2). Nevertheless, the most striking finding was that further on (over 40 passages) a fitness recovery was observed (Fig. 4), in both the population adapting from 0 to 0.05 µg/ml (lineage 1) and the population adapting from 0.05 to 0.2 µg/ml of AMD (lineage 2). In contrast, PV did not suffer a decrease in fitness during the process of adaptation to AMD and rather experienced a significant increase of virus production per cell when growing in the presence of 0.2 µg/ml of AMD (Fig. 3). However, PV follows a completely different strategy with a highly optimized codon usage to that of the cell and thus confirming that viral production is at its best when there is a good match between codon usage (demand) and tRNA availability (supply).

The analysis of the HAV codon usage adaptation to AMD revealed an interesting adjustment in the capsid region. If translation selection is the driving force of the codon usage, a tendency to optimize the codon usage should be expected. Instead what was detected was a re-deoptimization getting to an increased use of those uncommon tRNAs. This re-deoptimization was associated with a fitness recovery in terms of infectious virus production, suggesting that the loss of efficiency in translation would be compensated by a different capsid folding, affecting stability and/or exposure of the receptor binding site. Preliminary data point to a change in the antigenic structure and thermal stability of the viral capsid during the adaptation to AMD (data not shown). Further proteomic analyses to confirm these hypotheses are in progress, but the genomic studies provide evidence suggesting that fine-tuning translation selection is actually contributing to the codon usage bias of HAV. Additional selective pressures may derive from the decrease of some cellular factors required for HAV replication and translation in conditions of cellular shut-off. Virus adjustment to this new situation may be mediated by mutations inducing changes in the RNA structure. However, it is unlikely that these mutations result in a change in RNA structure concomitant with a change in codon usage. Other alternative interpretations include AMD-associated alterations of 3D, 3C or 3CD activities although fitness recovery was not associated with mutations in these regions.

Fine-tuning translation of the capsid is graphically evidenced during the back adaptation process of viral lineage 3, from 0.05 µg/ml to 0.0 µg/ml of AMD (Fig. S2), where the population seeks a kind of dynamic equilibrium regarding the variation in the anticodon usage. Most rare codons in the capsid coding region are rare codons pairing with abundant tRNAs, while only a few of them are rare codons pairing with rare tRNAs. Many of these rare codons pairing with abundant tRNAs (62%) were replaced during the process of adaptation to AMD, all of them to codons pairing with less abundant tRNAs, while those more common codons pairing with non-abundant tRNAs were replaced at a lower frequency (34%), and most of them to codons pairing with even less common tRNAs (Table S2). Most HAV capsid residues coded by rare codons are strategically located in the carboxy terminal regions of the putative highly structured elements [23]. A higher tendency of replacement of these strategically located rare codons in comparison with those located apart from these regions was observed (Table S3), indicating the potential relevance of the translation kinetics in providing locally slow ribosome traffic rates and thus contributing to the proper capsid protein folding. Actually, it is interesting that whereas the capsid folding of many picornaviruses is dependent on the activity of the heat-shock protein 90 (Hsp90) chaperone [35], that of HAV is not (Fig. 9). Additionally, the fact that substitutions detected in the 3D region during the adaptation to AMD do not tend to re-deoptimize the codon usage as occurs in the capsid region (Fig. 6) together with a significantly lower mutation rate in the 5′ NCR of the population adapting to the drug (data not shown), which are not under the pressure of the translational machinery, re-enforces the critical role of translation kinetics in the capsid region.

Selection for fine-tuning translation kinetics in the HAV capsid acts on the whole virus population and the flattest population (mutant spectrum) rather than the fittest individual is selected. In fact, a blend of mutations occurring around the swarm of genomes affecting the overall codon usage was associated with fitness recovery. The critical role of the mutant spectra is also observed in the faster adaptation of the populations during the back processes from higher to lower and from presence to absence of AMD than during the forward processes, pointing to the existence of molecular memory in the quasispecies as described elsewhere [36].

Codon usage adaptation to tRNA availability must find a critical balance between the rate of translation and the proper protein folding to reach the highest fitness. While, generally, the viral populations adapted to a given tRNA pool, out-competed the non-adapted populations under those specific conditions (Fig. 8), the exception to the rule was the particular case of lineage 2 (adapted to grow in 0.2 µg/ml of AMD) in competition experiments with lineage 1 (adapted to grow in 0.05 µg/ml of AMD) in the presence of 0.05 µg/ml of AMD. Under these conditions, the populations were unable to out-compete each other, suggesting some kind of cooperation rather than competition (Fig. 8 C and D). Although difficult to predict, it may be hypothesized that the expected slower translation rate of lineage 2 might be compensated with a higher quality capsid-folding and that the faster translation rate of lineage 1 might provide a higher level of the viral enzymes required for RNA replication and capsid maturation. Experiments are, presently, in progress to assess this point.

HAV behavior in terms of mutation-selection for a fine-tuning translation kinetics allowed for fitness recovery but not fitness gain during the different processes of adaptation to the cellular tRNA changing conditions and thus it may represent an additional view of the Red Queen dynamics of protein translation [37]. At least from the viral side it is clear that in a hostile environment “it takes all the running you can do to keep in the same place”. Evolutionary adaptive changes are required to maintain fitness and cessation of change may result in extinction.

## Materials and Methods

### Cells and viruses

The cytopathogenic pHM175 43c strain of HAV was used for the study of HAV replication and evolution in the presence of actinomycin D (AMD, Sigma). Serial passages in 0.0 µg/ml (lineage 0), 0.05 µg/ml (lineage 1) and 0.2 µg/ml (lineage 2) of AMD were carried out with a multiplicity of infection (m.o.i.) of 1, at a 7-day interval. Additionally, pre-adapted populations were returned to the original conditions from 0.05 µg/ml to 0.0 µg/ml of AMD (lineage 3) and from 0.2 µg/ml to 0.05 µg/ml of AMD (lineage 4). The LSc 2ab strain of poliovirus was also grown in the absence or presence of AMD and passaged every 2 days at a m.o.i. of 1. The infectious virus titer (TCID_50_) was obtained for both viruses in FRhK-4 cell monolayers. Virus yield per cell was figured taking in consideration the average cell viability under each condition (between days 4 and 7, and at day 2, for HAV and PV, respectively). HAV and PV production in the presence of geldadamycin concentrations from 0.062 to 1 µM was evaluated in the same way.

### Actinomycin D-induced cellular shut-off

AMD-associated cytotoxicity was measured by counting viable cells using the trypan blue exclusion method. Total cytoplasmic RNA abundance from 10^6^ cells treated with 0.2 µg/ml, 0.05 µg/ml or 0.0 µg/ml of AMD, and from untreated cells infected with HAV was quantified using the NanoDrop® ND-1000 spectrophotometer, as a measure of the cellular genome expression. Additionally the expression level of two cellular genes, HPRT-I (hypoxanthine phosphoribosyl-transferase I) and GAPDH (glyceraldehide-3-phosphate dehydrogenase) [38],[39], was also monitored by end-point dilution RT-PCR using previously described primers [40],[41] for all aforementioned conditions.

### Quasispecies analysis

Two genomic regions of the capsid coding region were analyzed: a fragment of the VP3-coding region, corresponding to amino acids 1–123, and a fragment within the VP1-coding region, corresponding to amino acids 85–245. Besides, another fragment corresponding to amino acids 1–253 of the nonstructural protein 3D (polymerase), was also analyzed. RT-PCR amplification of the specified RNA fragments was performed as described elsewhere [33]. Previously described primers [33] were used for the amplification of the VP3 and VP1 fragments, while for the 3D- fragment primers 3D- (5′ATGATTCTACCTGCTTCTCT3′) and 3CD (5′ATTGGGATCCAAGAAAATTGAAAGTCA3′) were designed. PCR products were cloned and the sequence from 50 molecular clones obtained as previously described [33].

### Genomic analysis

Viral codon usage tables were obtained for each viral population at several passages through the analysis of the sequences of 50 molecular clones. Two codon usage tables were made, one using the sequences of the VP1- and VP3-fragments as a model for the structural proteins coding region and another using the sequences of the 3D-region as a model for the non-structural proteins coding region. Anticodon usage tables were inferred from these codon usage tables by assuming a model based on the frequency of the codons, the anticodon degeneracy and the codon:anticodon match pairing preferences [19] (Tables S4 and S5). Additionally, an anticodon usage table was also drawn for the host cell (Tables S4 and S5) and anticodons sorted in those used less than 20%, those used between 20 and 60% and those used more than 60%. The relative variation of usage of each anticodon at each viral passage compared to the initial passage was calculated. The mean variation of the viral anticodons belonging to each of the previously defined groups was calculated and significant differences between the mean variations in the populations growing in the absence or presence of the drug analyzed by a T-student test.

### Growth competition experiments

Growth competition experiments between viral lineages 0 and 1 were performed after mixing the populations at ratios of 1∶1, 100∶1 and 1∶100 and grown in the absence or presence of 0.05 µg/ml of AMD. Additionally, competition experiments between lineages 1 and 2 at ratios 1∶1, 100∶1 and 1∶100, and grown in the presence of 0.05 µg/ml and 0.2 µg/ml of AMD were also performed. A m.o.i. of 1 was used, with the exception of those experiments in which the mixing ratios were 1∶1 in which the m.o.i. was 2. Viral progeny of each competition experiment was passaged several times and consensus sequences obtained.

To follow up the proportion of each population several genetic markers were used: two mutations that were present in the consensus sequence of lineage 1 and absent in lineage 0 (a→g at nucleotide 2459 and a→g at nucleotide 2643), and two additional mutations only present in lineage 2 (c→u at position 1282 and c→u at nucleotide 1393). These latter mutations occurred in the VP0 coding region which was sequenced using previously described primers [42]. These specific genetic markers allowed a semiquantitative monitoring of the populations through determination of the proportional height of the two peaks at each nucleotide position inferred from the chromatogram of the consensus sequences.

## Supporting Information

Table S1 Relative codon deoptimization indexes (RCDI) of different picornaviruses were calculated from the complete genome sequences available at the GenBank. The RCDI of the mutant spectra of the pHM175 43c strain of HAV growing in the absence or in the presence of 0.05 µg/ml and 0.2 µg/ml of AMD was also assessed.
(0.01 MB PDF)

Table S2 Number and percentage of codons replaced in the capsid region during both the process of adaptation to increasing concentrations of actinomycin D (passages P4, P5, P20, P36, P38, P41, P44, P65 and P85 of lineage 1 and P20 and P38 of lineage 2 were analyzed) and the re-adaptation to the absence of actinomycin D (passage P21 of lineage 3 was analyzed). The repetitions observed in different passages were not quantified. Codons were sorted between rare and common, being the rare codons those pairing with abundant tRNAs and the common codons those pairing with less abundant tRNAs. Those rare codons pairing with rare tRNAs were excluded of the analysis. As a control the substitutions detected in the population growing in the absence of the drug were also included (passages P4, P5, P20, P36, P38, P41, P44, P65 and P85 of lineage 1 were analyzed).
(0.01 MB PDF)

Table S3 Location of the rare codons pairing with abundant tRNAs in the VP3 and VP1 regions analyzed. The protein fragments were divided in two regions, the carboxy-ends and borders of the highly structured elements (β-barrels and α-helices) and the remaining part of the protein, following previously published criteria [23]. The so-called carboxy-limits were defined as the third carboxy-part of the structural elements plus the 5 contiguous external residues. In some instances, when the β-barrels or the α-helices were shorter than 3 residues, only one internal residue was included; when the length of the external joining residues was shorter than 5 before entering the next structural element, the totality of the joining region was included.
(0.01 MB PDF)

Table S4 Conversion from codon usage tables to anticodon usage variation in the capsid regions studied. A) Codon counts in 50 molecular clones of the different populations studied. B) Anticodon usage estimation from the codon counts, applying the multiple codon:anticodon pairing and the codon:anticodon coupling efficiencies described elsewhere [2]. C) Usage of each anticodon (in percentage) out of the total usage of the anticodons bearing the same aminoacid. D) Variation (increasing or decreaing) of the relative usage of each anticodon from the initial passage.
(0.05 MB PDF)

Table S5 Conversion from codon usage tables to anticodon usage variation in the polymerase region studied. A) Codon counts in 50 molecular clones of the different populations studied. B) Anticodon usage estimation from the codon counts, applying the multiple codon:anticodon pairing and the codon:anticodon coupling efficiencies described elsewhere [19]. C) Usage of each anticodon (in percentage) out of the total usage of the anticodons bearing the same aminoacid. D) Variation (increasing or decreaing) of the relative usage of each anticodon from the initial passage.
(0.03 MB PDF)

Figure S1 Infectious HAV titer production per cell during the first 20 adaptation passages of lineages 1 (adaptation to 0.05 µg/ml of AMD) and 3 (adaptation from 0.05 µg/ml to 0.0 µg/ml of AMD), and lineages 2 (adaptation from 0.05 µg/ml to 0.2 µg/ml of AMD) and 4 (adaptation from 0.2 µg/ml to 0.05 µg/ml of AMD). Linear regression analysis showed that the kinetics of adaptation were faster (with significant differences in the slopes, p<0.05) in the re-adaptation to the original conditions than in the first adaptation to the different AMD concentrations.
(0.99 MB TIF)

Figure S2 Re-adaptation from 0.05 to 0.0 µg/ml of AMD. Right panel depicts the behavior of the population re-adapting from 0.05 to 0.0 µg/ml of AMD (lineage 3). Left panel depicts the behavior of the population in 0.05 µg/ml of AMD as baseline control. The percentage of variation of each individual viral anticodon in the mutant spectra with respect to the cellular usage is shown. Mean and standard deviation of those anticodons with the highest level of variation (encircled) is shown.
(0.58 MB TIF)

Figure S3 Relative codon deoptimization index (RCDI) during the process of adaptation to AMD. A. RCDI (mean±SD) in the capsid coding region. B. RCDI (mean±SD) in the polymerase coding region. During the process of adaptation to 0.05 µ g/ml of AMD no differences were detected until P44 (hence two values are given). The first value represents passages P1-P44 and the second passages P45–P103. As it can be observed, the capsid region showed a significant increase of the RCDI during the process of adaptation to increasing AMD concentrations. On the contrary, the RCDI of the polymerase coding region although showing a tendency to decrease did not significantly vary.
(0.49 MB TIF)
