# Supplementary material for: Fine-Tuning Translation Kinetics Selection as the Driving Force of Codon Usage Bias in the Hepatitis A Virus Capsid
Source: PLoS Pathog. 2010 Mar 5;6(3):e1000797. doi: 10.1371/journal.ppat.1000797 (PMC2832697; doi:10.1371/journal.ppat.1000797)
Supplement: Table S1 — Relative codon deoptimization indexes (RCDI) of different picornaviruses were calculated from the complete genome sequences available at the GenBank. The RCDI of the mutant spectra of the pHM175 43c strain of HAV growing in the absence or in the presence of 0.05 µg/ml and 0.2 µg/ml of AMD was also assessed. (0.01 MB PDF) [file ppat.1000797.s001.pdf]

**Virus***	Capsid	Non-Structural Proteins	Whole Genome
PV-I	-	-	1.14
CV-B3	-	-	1.16
RhV-2	-	-	1.37
RhV-14	-	-	1.39
Aichi	-	-	1.39
HAV	-	-	1.70
HAV 0.0	1.732	1.613	1.635
HAV 0.05	1.740	1.614	1.637
HAV 0.2	1.745	1.608	1.635

* PV: poliovirus, CV: coxsackievirus, RhV: rhinovirus, Aichi: aichivirus, HAV: hepatitis A virus
