# Supplementary material for: Fine-Tuning Translation Kinetics Selection as the Driving Force of Codon Usage Bias in the Hepatitis A Virus Capsid
Source: PLoS Pathog. 2010 Mar 5;6(3):e1000797. doi: 10.1371/journal.ppat.1000797 (PMC2832697; doi:10.1371/journal.ppat.1000797)
Supplement: Table S2 — Number and percentage of codons replaced in the capsid region during both the process of adaptation to increasing concentrations of actinomycin D (passages P4, P5, P20, P36, P38, P41, P44, P65 and P85 of lineage 1 and P20 and P38 of lineage 2 were analyzed) and the re-adaptation to the absence of actinomycin D (passage P21 of lineage 3 was analyzed). The repetitions observed in different passages were not quantified. Codons were sorted between rare and common, being the rare codons those pairing with abundant tRNAs and the common codons those pairing with less abundant tRNAs. Those rare codons pairing with rare tRNAs were excluded of the analysis. As a control the substitutions detected in the population growing in the absence of the drug were also included (passages P4, P5, P20, P36, P38, P41, P44, P65 and P85 of lineage 1 were analyzed). (0.01 MB PDF) [file ppat.1000797.s002.pdf]

Type of codons	Viral Populations growing in the absence of AMD		Viral Populations adapting to AMD		Viral Populations adapted to AMD and re-adapting to absence of the drug	
Rare codons	14/34 (41%)	1 (7%)*	21/34 (62%)	0 (0%)*	6/34 (18%)	2 (33%)*
		5 (36%)†		0 (0%)†		1 (17%)†
		8 (57%)‡		21 (100%)‡		3 (50%)‡
Common codons	69/252 (27%)	30 (43%)*	86/252 (34%)	15 (18%)*	15/252 (6%)	9(60%)*
		17 (25%)†		20 (23%)†		4 (27%)†
		22 (32%)‡		51 (59%)‡		2 (13%)‡

*The new generated codon paired with a more common tRNA than the tRNA pairing with the original codon.

†The new generated codon paired with a tRNA similarly common than the tRNA pairing with the original codon.

‡The new generated codon paired with a less common tRNA than the tRNA pairing with the original codon.
