# Supplementary material for: Fine-Tuning Translation Kinetics Selection as the Driving Force of Codon Usage Bias in the Hepatitis A Virus Capsid
Source: PLoS Pathog. 2010 Mar 5;6(3):e1000797. doi: 10.1371/journal.ppat.1000797 (PMC2832697; doi:10.1371/journal.ppat.1000797)
Supplement: Table S3 — Location of the rare codons pairing with abundant tRNAs in the VP3 and VP1 regions analyzed. The protein fragments were divided in two regions, the carboxy-ends and borders of the highly structured elements (β-barrels and α-helices) and the remaining part of the protein, following previously published criteria [23]. The so-called carboxy-limits were defined as the third carboxy-part of the structural elements plus the 5 contiguous external residues. In some instances, when the β-barrels or the α-helices were shorter than 3 residues, only one internal residue was included; when the length of the external joining residues was shorter than 5 before entering the next structural element, the totality of the joining region was included. (0.01 MB PDF) [file ppat.1000797.s003.pdf]

Genomic Region	Location	Total number of rare codons	Number of mutated rare codons (% of the total)
VP3	COOH borders	6	5 (83)
	Rest of the protein	5	2 (40)
VP1	COOH borders	11	7 (64)
	Rest of the protein	12	7 (58)
Total	COOH borders	17	12 (71)
	Rest of the protein	17	9 (53)
