# Supplementary material for: Fine-Tuning Translation Kinetics Selection as the Driving Force of Codon Usage Bias in the Hepatitis A Virus Capsid
Source: PLoS Pathog. 2010 Mar 5;6(3):e1000797. doi: 10.1371/journal.ppat.1000797 (PMC2832697; doi:10.1371/journal.ppat.1000797)
Supplement: Table S4 — Conversion from codon usage tables to anticodon usage variation in the capsid regions studied. A) Codon counts in 50 molecular clones of the different populations studied. B) Anticodon usage estimation from the codon counts, applying the multiple codon:anticodon pairing and the codon:anticodon coupling efficiencies described elsewhere [2]. C) Usage of each anticodon (in percentage) out of the total usage of the anticodons bearing the same aminoacid. D) Variation (increasing or decreaing) of the relative usage of each anticodon from the initial passage. (0.05 MB PDF) [file ppat.1000797.s004.pdf]

Table S4. Conversion from codon usage tables to anticodon usage variation in the capsid regions studied. A) Codon counts in 50 molecular clones of the different populations studied. B) Anticodon usage estimation from the codon counts, applying the multiple codon:anticodon pairing and the codon:anticodon coupling efficiencies described in (dos Reis M, Savva R. Wernisch L (2004) Solving the riddle of codon usage preferences: a test for tranlational selection. Nucl Acids Res 32:5036-5044) C) Usage of each anticodon (in percentage) out of the total usage of the anticodons bearing the same aminoacid. D) Variation (increasing or decreaing) of the relative usage of each anticodon from the initial passage.

**A) Codon usage**		0.0 µg/ml AMD											0.05 µg/ml AMD									0.2 µg/ml AMD		
		P4	P5	P20	P36	P38	P41	P44	P65	P85	P103	P21/R	P4	P5	P20	P36	P38	P41	P44	P65	P85	P20	P38	Cell
Val	GUU	309	303	307	306	319	305	310	306	318	316	323	301	304	309	321	338	334	344	336	347	346	376	34
	GUG	150	150	154	150	150	150	150	150	150	150	150	150	150	150	150	150	150	150	149	151	151	150	100
	GUA	150	150	150	150	150	151	150	152	150	151	149	150	150	151	150	150	150	150	151	150	148	124	19
	GUC	153	150	154	152	154	145	152	156	158	150	175	151	151	155	162	189	187	187	187	187	200	199	53
Ile	AUU	643	646	645	644	632	647	643	644	637	636	623	648	647	641	639	613	615	609	614	608	604	600	61
	AUA	100	100	100	100	101	100	101	100	100	101	100	100	100	100	100	100	100	100	100	101	104	100	24
	AUC	96	102	94	98	94	95	94	92	75	97	74	99	99	95	85	60	63	58	60	38	46	51	100
Ser	UCU	700	700	700	700	700	699	700	700	698	700	700	700	698	700	700	700	702	699	694	698	699	702	71
	UCA	351	349	350	350	349	350	350	350	351	350	350	349	350	350	350	350	346	345	352	351	348	350	50
	AGU	50	50	50	50	51	50	50	50	50	50	49	50	50	50	50	50	51	50	50	51	50	50	50
	UCC	100	100	100	100	100	101	101	100	101	100	100	100	101	100	98	89	98	100	100	100	101	99	95
	UCG	0	1	0	0	0	1	0	0	1	0	0	1	3	0	0	1	1	0	2	0	2	0	22
	AGC	26	35	28	25	27	29	27	26	29	30	46	34	35	10	11	20	25	19	27	20	46	50	100
Leu	UUG	599	600	599	600	600	599	598	602	595	583	598	599	600	600	601	600	600	600	606	582	600	599	26
	UUA	150	151	150	150	150	150	150	150	150	150	150	151	150	150	149	150	154	156	150	151	150	151	12
	CUU	151	150	150	150	150	150	150	150	151	150	150	152	153	150	150	149	150	151	150	150	151	150	25
	CUG	201	200	201	200	201	200	201	198	199	199	200	201	200	200	200	202	197	199	192	199	200	200	100
	CUA	0	0	1	0	0	0	0	0	0	0	0	0	1	0	0	0	0	1	7	1	0	0	15
	CUC	0	0	0	0	0	0	0	0	0	0	0	0	0	0	0	0	0	0	0	0	0	0	47
Gly	GGA	300	300	300	300	301	300	300	303	300	300	300	300	300	300	300	300	300	300	305	300	300	298	67
	GGU	349	351	350	349	348	350	350	343	350	351	350	350	351	350	350	350	351	350	337	350	351	349	44
	GGG	152	150	150	152	150	172	182	150	151	150	187	150	150	150	150	150	150	150	149	149	150	150	68
	GGC	50	49	50	50	50	50	50	57	50	50	51	50	50	50	50	49	49	50	59	50	48	50	100
Asn	AAU	651	650	650	650	650	648	654	651	664	650	650	650	650	650	651	650	650	651	657	651	654	651	73
	AAC	99	100	100	100	102	102	102	101	99	100	100	100	100	100	99	100	100	100	97	100	97	101	100
Cys	UGU	151	151	150	150	150	150	151	150	150	151	150	150	150	151	150	151	150	150	153	150	150	150	68
	UGC	50	50	50	50	50	50	50	50	51	50	50	50	50	50	50	50	50	50	50	50	50	50	100
Thr	ACU	600	599	600	600	599	600	598	600	600	599	602	599	598	601	601	599	599	600	600	601	600	600	55
	ACA	549	550	550	550	550	549	549	553	550	530	546	550	550	550	550	549	551	550	549	551	549	551	63
	ACC	123	115	122	125	125	122	123	119	119	117	155	116	113	139	138	111	106	111	101	120	100	100	100
	ACG	1	0	0	0	0	0	0	0	0	0	0	0	0	0	0	2	0	0	0	0	1	0	29
Pro	CCU	300	300	300	300	300	301	299	300	298	300	301	300	300	299	301	301	300	300	300	299	300	300	78
	CCA	346	349	348	349	350	349	352	350	350	350	373	349	341	350	351	350	349	348	345	350	350	349	73
	CCC	50	50	50	50	50	50	50	50	50	50	50	51	50	50	50	50	50	50	50	50	50	50	100
	CCG	55	51	51	52	51	47	48	50	50	50	26	49	60	50	49	49	53	51	49	51	50	50	33
Lys	AAA	350	350	350	350	350	350	350	347	350	350	349	350	347	350	350	350	350	350	352	349	364	349	64
	AAG	200	200	200	200	200	200	200	200	200	200	200	200	200	200	200	200	200	200	198	200	199	199	100
His	CAU	200	200	200	200	199	200	200	200	200	200	200	200	198	200	200	200	200	201	202	202	211	195	65
	CAC	1	0	0	0	4	3	0	5	7	0	16	1	0	0	5	0	0	1	1	1	0	5	100

**A) Codon usage**		0.0 µg/ml AMD											0.05 µg/ml AMD									0.2 µg/ml AMD		
		P4	P5	P20	P36	P38	P41	P44	P65	P85	P103	P21/R	P4	P5	P20	P36	P38	P41	P44	P65	P85	P20	P38	Cell
Phe	UUU	952	950	950	951	953	951	952	950	948	954	951	951	949	950	953	949	949	952	950	961	951	950	70
	UUC	248	250	250	250	247	249	249	250	260	266	251	249	250	250	247	250	251	249	251	267	249	249	100
Tyr	UAU	300	299	300	300	301	300	299	300	300	300	300	301	300	300	296	299	300	300	303	300	302	300	66
	UAC	249	250	250	250	245	247	250	245	246	250	234	249	250	250	250	250	250	249	249	246	250	250	100
Asp	GAU	650	649	649	650	650	648	645	647	635	650	650	650	651	650	650	653	650	649	643	650	648	650	75
	GAC	200	201	200	200	199	202	201	201	202	200	199	200	199	200	200	198	200	200	201	200	201	201	100
Glu	GAA	200	200	201	200	200	200	200	200	200	200	202	200	200	200	200	200	200	200	200	200	206	200	65
	GAG	150	150	150	150	150	150	150	155	150	150	158	150	150	150	150	150	150	151	150	150	164	152	100
Gln	CAA	301	300	300	300	300	300	300	297	300	300	300	299	300	300	300	300	299	300	300	300	301	309	33
	CAG	299	300	300	300	299	300	300	303	300	300	300	300	299	300	299	300	300	299	300	298	298	292	100
Ala	GCU	496	500	500	499	500	500	501	500	500	498	499	499	497	502	500	498	501	500	501	501	500	500	67
	GCA	250	250	250	250	250	249	250	250	250	249	250	250	250	249	250	250	250	250	249	248	250	250	48
	GCC	152	150	150	150	150	151	150	155	151	150	150	151	151	149	150	150	149	150	157	152	157	149	100
	GCG	50	50	50	49	50	28	18	45	50	51	14	50	50	50	50	51	50	49	52	51	50	48	25
Arg	AGA	350	350	350	350	350	350	350	350	350	350	350	350	350	350	350	350	350	350	350	346	333	352	88
	AGG	200	200	200	200	200	200	200	200	200	200	200	200	199	200	250	200	200	200	200	200	200	200	98
	CGC	0	0	0	0	0	0	0	0	0	0	0	0	0	0	0	0	0	0	0	0	0	0	100
	CGU	0	0	0	0	0	0	0	0	0	0	0	0	1	0	0	0	0	0	0	0	0	0	42
	CGA	0	0	0	0	0	0	0	0	0	0	0	0	0	0	0	0	1	0	0	0	0	0	48
	CGG	0	0	0	0	0	0	0	0	0	0	0	0	1	0	0	0	0	0	0	0	0	0	92

**B) Anticodon usage**		0.0 µg/ml AMD											0.05 µg/ml AMD									0.2 µg/ml AMD		
		P4	P5	P20	P36	P38	P41	P44	P65	P85	P103	P21/R	P4	P5	P20	P36	P38	P41	P44	P65	P85	P20	P38	Cell
Val	CAC	114	114	117	114	114	114	114	114	114	114	114	114	114	114	114	114	114	114	113	114	114	114	76
	CAU	206	206	207	206	206	207	206	208	206	207	205	206	206	207	206	206	206	206	206	206	204	180	56
	CAG	204	200	203	202	208	197	203	204	210	204	222	199	201	205	213	235	233	236	233	237	245	255	43
	CAI	64	63	65	64	65	61	64	65	66	63	73	63	63	65	68	79	78	78	78	78	84	83	22
	CAA	194	191	193	192	201	192	195	192	200	199	203	189	191	194	202	213	210	216	211	218	218	237	21
Ile	UAG	202	207	202	204	199	203	201	200	189	201	185	205	205	201	195	175	177	173	175	161	164	166	72
	UAI	288	292	288	290	283	290	288	287	277	286	271	292	291	287	282	262	264	259	262	251	252	253	65
	UAA	248	249	249	249	244	250	248	249	246	245	240	250	250	247	247	237	237	235	237	235	233	232	24
	UAU	100	100	100	100	101	100	101	100	100	101	100	100	100	100	100	100	100	100	100	101	104	100	24
Ser	AGA	270	270	270	270	270	270	270	270	269	270	270	270	269	270	270	270	280	280	278	279	280	271	27
	AGG	218	218	218	218	218	218	218	218	218	218	218	218	218	218	217	211	217	217	216	217	218	218	71
	AGI	312	312	312	312	312	312	313	312	312	312	312	312	312	312	311	308	312	312	310	311	312	312	67
	AGU	351	349	350	350	349	350	350	350	351	350	350	349	351	350	350	350	346	345	352	351	348	350	55
	UCA	19	19	19	19	20	19	19	19	19	19	19	19	19	19	19	19	20	19	19	20	19	19	19
	UCG	27	32	28	26	27	28	27	27	28	29	38	31	32	17	18	23	26	22	27	23	30	40	70
	UCI	30	34	31	30	31	31	31	30	31	32	38	34	34	23	24	28	30	27	31	28	30	40	61
	AGC	0	1	0	0	0	1	0	0	1	0	0	1	2	0	0	1	1	0	2	0	2	0	17
Leu	AAC	454	455	454	455	455	454	453	456	451	442	453	454	455	455	456	455	455	455	459	441	455	454	20
	AAU	295	296	295	295	295	295	295	296	294	291	295	296	295	295	294	295	299	301	297	292	295	296	18
	AAI	0	0	0	0	0	0	0	0	0	0	0	0	0	0	0	0	0	0	0	0	0	0	0
	GAA	58	58	58	58	58	58	58	58	58	58	58	59	59	58	58	58	58	58	58	58	58	58	10
	GAG	34	34	34	34	34	34	34	34	34	34	34	35	35	34	34	34	34	34	34	34	34	34	33
	GAI	58	58	58	58	58	58	58	58	58	58	58	59	59	58	58	58	58	58	58	58	58	58	29
	GAC	152	152	152	152	152	152	152	150	151	151	152	152	152	152	152	153	149	151	146	151	152	152	76
	GAU	49	48	50	48	49	48	49	48	48	48	48	49	49	48	48	49	48	49	53	49	48	48	39
Gly	CCU	337	336	336	337	337	342	344	339	337	336	345	336	336	336	336	336	336	336	341	336	336	334	83
	CCI	156	156	156	156	155	156	156	156	156	156	156	156	156	156	156	156	156	156	155	156	156	156	59
	CCA	135	135	135	135	134	135	135	132	135	135	135	135	135	135	135	135	135	135	130	135	135	135	17
	CCG	109	108	109	109	108	109	109	111	109	109	109	109	109	109	109	108	108	109	111	109	108	109	68
	CCC	115	114	114	115	114	130	138	114	114	114	142	114	114	114	114	114	114	114	113	113	114	114	51
Asn	UUA	251	251	251	251	251	250	252	251	256	251	251	251	251	251	251	251	251	251	254	251	252	251	28
	UUG	238	238	238	238	239	239	240	239	241	238	238	238	238	238	238	238	238	238	238	238	238	239	78
	UUI	293	293	293	293	294	293	295	294	298	293	293	293	293	293	293	293	293	293	294	293	293	294	70
Cys	ACA	58	58	58	58	58	58	58	58	58	58	58	58	58	58	58	58	58	58	59	58	58	58	26
	ACG	63	63	63	63	63	63	63	63	64	63	63	63	63	63	63	63	63	63	64	63	63	63	74
	ACI	79	79	79	79	79	79	79	79	79	79	79	79	79	79	79	79	79	79	80	79	79	79	68
Thr	UGA	232	231	232	232	231	232	231	232	232	231	232	231	231	232	232	231	231	232	232	232	232	232	21
	UGG	208	203	208	209	209	208	208	206	206	205	227	204	202	218	217	201	198	201	195	207	195	195	71
	UGI	462	462	462	462	462	462	461	463	462	453	461	462	461	462	462	461	462	462	462	463	462	462	48
	UGU	549	550	550	550	550	549	549	553	550	530	546	550	550	550	550	549	551	550	549	551	549	551	70
	UGC	0	0	0	0	0	0	0	0	0	0	0	0	0	0	0	2	0	0	0	0	1	0	22

B) Anticodon usage		0.0 µg/ml AMD											0.05 µg/ml AMD									0.2 µg/ml AMD		
		P4	P5	P20	P36	P38	P41	P44	P65	P85	P103	P21/R	P4	P5	P20	P36	P38	P41	P44	P65	P85	P20	P38	Cell
Pro	GGA	116	116	116	116	116	116	115	116	115	116	116	116	116	115	116	116	116	116	116	115	116	116	30
	GGG	97	97	97	97	97	97	97	97	97	97	97	98	97	97	97	97	97	97	97	97	97	97	76
	GGI	137	137	137	137	137	137	136	137	136	137	137	137	137	136	137	137	137	137	137	136	137	137	72
	GGU	359	361	360	362	362	360	364	362	362	362	379	361	355	362	363	362	362	360	357	362	362	361	81
	GGC	23	21	21	22	21	20	20	21	21	21	11	21	25	21	21	21	22	21	21	21	21	21	14
Lys	UUU	350	350	350	350	350	350	350	347	350	350	349	350	347	350	350	350	350	350	352	349	364	349	64
	UUC	200	200	200	200	200	200	200	200	200	200	200	200	200	200	200	200	200	200	198	200	199	199	100
	UUI	0	0	0	0	0	0	0	0	0	0	0	0	0	0	0	0	0	0	0	0	0	0	0
His	GUA	77	77	77	77	77	77	77	77	77	77	77	77	76	77	77	77	77	78	78	78	81	75	25
	GUG	46	46	46	46	48	47	46	49	50	46	55	46	45	46	49	46	46	46	47	47	48	47	73
	GUI	78	77	77	77	78	78	77	79	80	77	84	78	76	77	79	77	77	78	78	78	81	77	67
Phe	AAA	367	367	367	367	368	367	367	367	366	368	367	367	366	367	368	366	366	367	367	371	367	367	27
	AAG	361	362	362	362	361	361	362	362	367	372	363	361	362	362	361	362	362	362	362	374	361	361	74
	AAI	471	471	471	472	471	471	472	471	475	480	472	471	471	471	471	471	471	472	472	483	471	471	69
Tyr	AUA	116	115	116	116	116	116	115	116	116	116	116	116	116	116	114	115	116	116	117	116	117	116	25
	AUG	213	213	214	214	211	212	213	211	211	214	204	213	214	214	213	213	214	213	214	211	214	214	73
	AUI	220	220	221	221	219	219	220	218	219	221	214	221	221	221	219	220	221	220	221	219	221	221	67
Asp	CUA	251	251	251	251	251	250	249	250	245	251	251	251	251	251	251	252	251	251	248	251	250	251	29
	CUG	264	265	264	264	264	265	264	264	262	264	264	264	264	264	264	264	264	264	263	264	265	265	75
	CUI	335	335	334	335	334	335	333	334	330	335	334	335	335	335	335	335	335	334	332	335	334	335	71
Glu	CUU	236	236	237	236	236	236	236	237	236	236	240	236	236	236	236	236	236	237	236	236	246	237	89
	CUI	0	0	0	0	0	0	0	0	0	0	0	0	0	0	0	0	0	0	0	0	0	0	0
	CUC	112	112	112	112	112	112	112	116	112	112	118	112	112	112	112	112	112	113	112	112	123	114	75
Gln	GUU	373	373	373	373	372	373	373	370	373	373	373	372	372	373	372	373	372	372	373	372	373	380	57
	GUI	0	0	0	0	0	0	0	0	0	0	0	0	0	0	0	0	0	0	0	0	0	0	0
	GUC	227	227	227	227	227	227	227	230	227	227	227	227	227	227	227	227	227	227	227	226	226	221	76
Ala	CGA	191	193	193	193	193	193	193	193	193	192	193	193	192	194	193	192	193	193	193	193	193	193	26
	CGG	226	226	226	226	226	227	226	229	227	226	226	226	226	226	226	226	226	226	230	228	230	226	77
	CGI	255	256	256	255	256	256	256	258	256	255	255	256	255	256	256	255	256	256	259	257	259	255	68
	CGU	262	262	262	262	262	256	254	261	262	261	253	262	262	261	262	262	262	262	262	260	262	262	54
	CGC	38	38	38	37	38	21	14	34	38	39	11	38	38	38	38	39	38	37	39	39	38	36	19
Arg	UCU	424	424	424	424	424	424	424	424	424	424	424	424	424	424	442	424	424	424	424	420	407	426	124
	UCI	0	0	0	0	0	0	0	0	0	0	0	0	0	0	0	0	0	0	0	0	0	0	0
	UCC	126	126	126	126	126	126	126	126	126	126	126	126	125	126	157	126	126	126	126	126	126	126	62
	GCG	0	0	0	0	0	0	0	0	0	0	0	0	0	0	0	0	0	0	0	0	0	0	68
	GCI	0	0	0	0	0	0	0	0	0	0	0	0	0	0	0	0	0	0	0	0	0	0	58
	GCA	0	0	0	0	0	0	0	0	0	0	0	0	0	0	0	0	0	0	0	0	0	0	16
	GCU	0	0	0	0	0	0	0	0	0	0	0	0	0	0	0	0	1	0	0	0	0	0	70
	GCC	0	0	0	0	0	0	0	0	0	0	0	0	1	0	0	0	0	0	0	0	0	0	70

C) Relative anticodon usage		0.0 µg/ml AMD											0.05 µg/ml AMD									0.2 µg/ml AMD		
		P4	P5	P20	P36	P38	P41	P44	P65	P85	P103	P21/R	P4	P5	P20	P36	P38	P41	P44	P65	P85	P20	P38	Cell
Val	CAU	26,32	26,62	26,39	26,45	25,95	26,82	26,32	26,51	25,85	26,28	25,07	26,66	26,56	26,34	25,63	24,30	24,47	24,18	24,49	24,11	23,60	20,69	74
	CAC	14,55	14,72	14,87	14,63	14,35	14,76	14,55	14,51	14,30	14,46	13,93	14,74	14,68	14,50	14,17	13,43	13,53	13,37	13,41	13,40	13,24	13,09	100
	CAG	26,05	25,84	25,91	25,96	26,23	25,62	26,02	26,06	26,38	25,99	27,13	25,85	25,89	26,10	26,57	27,79	27,67	27,79	27,70	27,78	28,29	29,38	57
	CAI	8,21	8,14	8,22	8,19	8,15	7,89	8,15	8,35	8,33	7,99	8,98	8,20	8,17	8,28	8,46	9,36	9,33	9,22	9,30	9,17	9,70	9,60	29
	CAA	24,88	24,68	24,60	24,76	25,32	24,90	24,96	24,57	25,15	25,28	24,89	24,55	24,69	24,78	25,16	25,12	25,00	25,45	25,09	25,54	25,18	27,24	28
Ile	UAI	34,38	34,45	34,37	34,40	34,26	34,39	34,32	34,35	34,15	34,31	34,06	34,43	34,42	34,36	34,26	33,86	33,91	33,82	33,87	33,55	33,48	33,69	91
	UAG	24,12	24,36	24,04	24,20	24,03	24,07	24,01	23,96	23,25	24,14	23,22	24,23	24,24	24,08	23,67	22,59	22,73	22,50	22,59	21,51	21,81	22,16	100
	UAA	29,58	29,41	29,67	29,52	29,50	29,66	29,62	29,73	30,28	29,44	30,17	29,53	29,52	29,60	29,93	30,61	30,51	30,65	30,62	31,42	30,92	30,84	33
	UAU	11,92	11,79	11,92	11,88	12,21	11,88	12,05	11,96	12,31	12,11	12,55	11,81	11,82	11,96	12,13	12,94	12,85	13,04	12,92	13,52	13,79	13,31	33
Ser	AGA	22,02	21,88	22,00	22,06	22,02	21,94	22,00	22,04	21,90	21,97	21,70	21,90	21,78	22,33	22,35	22,33	22,73	22,89	22,51	22,69	22,59	21,66	38
	AGG	17,74	17,63	17,73	17,77	17,74	17,73	17,78	17,76	17,71	17,70	17,49	17,64	17,61	17,99	17,91	17,46	17,61	17,78	17,52	17,67	17,59	17,39	100
	AGI	25,44	25,27	25,42	25,48	25,44	25,38	25,45	25,46	25,35	25,38	25,07	25,29	25,20	25,80	25,75	25,42	25,33	25,49	25,08	25,32	25,19	24,98	94
	AGU	28,60	28,28	28,50	28,57	28,44	28,47	28,50	28,55	28,55	28,45	28,11	28,30	28,35	28,92	28,95	28,94	28,10	28,20	28,54	28,54	28,11	27,97	77
	UCA	1,57	1,56	1,57	1,58	1,60	1,57	1,57	1,57	1,57	1,57	1,52	1,56	1,56	1,60	1,60	1,60	1,60	1,58	1,56	1,60	1,56	1,54	27
	UCG	2,16	2,57	2,25	2,12	2,23	2,30	2,21	2,16	2,30	2,34	3,04	2,52	2,57	1,42	1,47	1,90	2,12	1,83	2,19	1,89	2,42	3,23	97
	UCI	2,46	2,75	2,53	2,43	2,53	2,56	2,49	2,46	2,56	2,59	3,07	2,72	2,75	1,94	1,98	2,29	2,45	2,23	2,48	2,28	2,42	3,22	86
	AGC	0	0,06	0,00	0,00	0,00	0,06	0,00	0,00	0,06	0,00	0,00	0,06	0,18	0,00	0,00	0,06	0,06	0,00	0,12	0,00	0,12	0,00	23
Leu	AAC	41,24	41,31	41,24	41,35	41,31	41,32	41,25	41,49	41,19	40,85	41,29	41,17	41,20	41,35	41,42	41,31	41,31	41,09	41,58	40,74	41,31	41,28	26
	AAU	26,79	26,91	26,79	26,84	26,81	26,84	26,82	26,88	26,85	26,90	26,84	26,83	26,74	26,84	26,77	26,81	27,18	27,21	26,85	26,95	26,81	26,91	24
	AAI	0,00	0,00	0,00	0,00	0,00	0,00	0,00	0,00	0,00	0,00	0,00	0,00	0,00	0,00	0,00	0,00	0,00	0,00	0,00	0,00	0,00	0,00	0
	GAA	5,29	5,26	5,26	5,26	5,26	5,27	5,27	5,26	5,32	5,35	5,27	5,32	5,35	5,26	5,26	5,22	5,26	5,27	5,24	5,35	5,29	5,26	13
	GAG	3,11	3,09	3,09	3,10	3,09	3,10	3,10	3,10	3,13	3,15	3,10	3,13	3,15	3,10	3,10	3,07	3,09	3,10	3,08	3,14	3,11	3,10	44
	GAI	5,29	5,26	5,26	5,26	5,26	5,27	5,27	5,26	5,32	5,35	5,27	5,32	5,35	5,26	5,26	5,22	5,26	5,27	5,24	5,35	5,29	5,26	39
	GAC	13,84	13,77	13,84	13,78	13,84	13,80	13,87	13,65	13,78	13,94	13,81	13,81	13,73	13,78	13,78	13,91	13,56	13,63	13,17	13,93	13,77	13,78	100
	GAU	4,42	4,40	4,51	4,40	4,42	4,40	4,43	4,36	4,40	4,45	4,41	4,41	4,48	4,40	4,40	4,44	4,33	4,44	4,84	4,54	4,40	4,40	52
Gly	CCU	39,58	39,57	39,57	39,58	39,73	39,18	39,01	39,78	39,55	39,52	38,88	39,57	39,52	39,57	39,57	39,61	39,57	39,57	40,13	39,59	39,61	39,47	100
	CCI	18,30	18,36	18,37	18,30	18,30	17,90	17,70	18,33	18,34	18,39	17,63	18,37	18,39	18,37	18,37	18,34	18,36	18,37	18,22	18,39	18,33	18,39	71
	CCA	15,83	15,94	15,90	15,83	15,82	15,50	15,32	15,52	15,88	15,92	15,22	15,90	15,92	15,90	15,90	15,92	15,94	15,90	15,31	15,92	15,96	15,91	20
	CCG	12,77	12,77	12,81	12,77	12,77	12,49	12,34	13,05	12,79	12,82	12,33	12,81	12,82	12,81	12,81	12,75	12,77	12,81	13,07	12,82	12,71	12,83	82
	CCC	13,52	13,36	13,36	13,52	13,38	14,93	15,62	13,31	13,43	13,35	15,94	13,36	13,35	13,36	13,36	13,38	13,36	13,36	13,27	13,29	13,38	13,41	62
Asn	UUA	32,14	32,09	32,09	32,09	32,01	32,00	32,03	32,06	32,22	32,09	32,09	32,09	32,09	32,09	32,14	32,09	32,09	32,10	32,26	32,10	32,24	32,06	36
	UUG	30,42	30,46	30,46	30,46	30,53	30,54	30,51	30,49	30,35	30,46	30,46	30,46	30,46	30,46	30,42	30,46	30,46	30,45	30,32	30,45	30,33	30,49	100
	UUI	37,44	37,45	37,45	37,45	37,46	37,46	37,46	37,45	37,43	37,45	37,45	37,45	37,45	37,45	37,44	37,45	37,45	37,45	37,43	37,45	37,43	37,45	89
Cys	ACA	29,00	29,00	28,95	28,95	28,95	28,95	29,00	28,95	28,81	29,00	28,95	28,95	28,95	29,00	28,95	29,00	28,95	28,95	29,09	28,95	28,95	28,95	36
	ACG	31,58	31,58	31,63	31,63	31,63	31,63	31,58	31,63	31,76	31,58	31,63	31,63	31,63	31,58	31,63	31,58	31,63	31,63	31,49	31,63	31,63	31,63	100
	ACI	39,42	39,42	39,43	39,43	39,43	39,43	39,42	39,43	39,44	39,42	39,43	39,43	39,43	39,42	39,43	39,42	39,43	39,43	39,41	39,43	39,43	39,43	93
Thr	UGA	15,96	15,99	15,96	15,94	15,92	15,97	15,94	15,93	15,98	16,29	15,84	15,98	15,98	15,87	15,87	16,01	16,03	16,03	16,11	15,97	16,10	16,08	30
	UGG	14,36	14,06	14,31	14,41	14,41	14,32	14,35	14,17	14,21	14,41	15,50	14,10	13,99	14,89	14,86	13,92	13,74	13,93	13,60	14,23	13,55	13,54	100
	UGI	31,82	31,92	31,84	31,80	31,80	31,84	31,82	31,87	31,88	31,94	31,44	31,91	31,94	31,63	31,64	31,93	32,04	31,98	32,11	31,87	32,10	32,12	67
	UGU	37,86	38,03	37,89	37,85	37,87	37,86	37,90	38,03	37,94	37,35	37,22	38,01	38,08	37,61	37,63	38,04	38,20	38,06	38,18	37,93	38,19	38,26	99
	UGC	0,00	0,00	0,00	0,00	0,00	0,00	0,00	0,00	0,00	0,00	0,00	0,00	0,00	0,00	0,00	0,10	0,00	0,00	0,00	0,00	0,05	0,00	31

C) Relative anticodon usage		0.0 µg/ml AMD											0.05 µg/ml AMD									0.2 µg/ml AMD		
		P4	P5	P20	P36	P38	P41	P44	P65	P85	P103	P21/R	P4	P5	P20	P36	P38	P41	P44	P65	P85	P20	P38	Cell
Pro	GGA	15,82	15,81	15,83	15,80	15,79	15,90	15,76	15,80	15,74	15,80	15,68	15,82	15,86	15,77	15,83	15,85	15,78	15,83	15,93	15,76	15,80	15,83	37
	GGG	13,27	13,26	13,28	13,25	13,25	13,33	13,23	13,26	13,23	13,26	13,14	13,35	13,30	13,25	13,27	13,28	13,24	13,28	13,36	13,23	13,26	13,28	94
	GGI	18,69	18,68	18,70	18,66	18,65	18,77	18,62	18,67	18,61	18,67	18,51	18,74	18,73	18,64	18,69	18,71	18,64	18,70	18,81	18,62	18,67	18,69	89
	GGU	49,08	49,33	49,26	49,32	49,40	49,31	49,64	49,41	49,55	49,41	51,19	49,29	48,67	49,48	49,42	49,36	49,31	49,26	49,08	49,47	49,41	49,34	100
	GGC	3,15	2,92	2,92	2,97	2,91	2,69	2,75	2,86	2,87	2,86	1,47	2,80	3,44	2,86	2,80	2,80	3,03	2,92	2,82	2,92	2,86	2,86	17
Lys	UUU	63,63	63,63	63,63	63,63	63,63	63,63	63,63	63,43	63,63	63,63	63,56	63,63	63,43	63,63	63,63	63,63	63,63	63,63	63,99	63,56	64,65	63,68	64
	UUC	36,36	36,36	36,36	36,36	36,36	36,36	36,36	36,56	36,36	36,36	36,43	36,36	36,56	36,36	36,36	36,36	36,36	36,36	36,00	36,43	35,35	36,31	100
	UUI	0,00	0,00	0,00	0,00	0,00	0,00	0,00	0,00	0,00	0,00	0,00	0,00	0,00	0,00	0,00	0,00	0,00	0,00	0,00	0,00	0,00	0,00	0
His	GUA	38,41	38,60	38,60	38,60	37,84	38,03	38,60	37,66	37,29	38,60	35,74	38,41	38,60	38,60	37,66	38,60	38,60	38,41	38,41	38,41	38,60	37,64	34
	GUG	22,98	22,80	22,80	22,80	23,50	23,32	22,80	23,66	23,99	22,80	25,41	22,98	22,80	22,80	23,66	22,80	22,80	22,97	22,97	22,97	22,80	23,68	100
	GUI	38,62	38,60	38,60	38,60	38,67	38,65	38,60	38,68	38,71	38,60	38,84	38,62	38,60	38,60	38,68	38,60	38,60	38,62	38,62	38,62	38,60	38,68	92
Phe	AAA	30,62	30,56	30,56	30,57	30,65	30,59	30,60	30,56	30,29	30,18	30,54	30,59	30,55	30,56	30,65	30,55	30,53	30,60	30,53	30,21	30,59	30,58	36
	AAG	30,10	30,15	30,15	30,15	30,07	30,12	30,12	30,15	30,40	30,50	30,17	30,12	30,16	30,15	30,07	30,16	30,18	30,12	30,18	30,48	30,12	30,13	100
	AAI	39,28	39,29	39,29	39,29	39,28	39,28	39,28	39,29	39,31	39,32	39,29	39,28	39,29	39,29	39,28	39,29	39,29	39,28	39,29	39,32	39,28	39,29	93
Tyr	AUA	21,09	21,02	21,05	21,05	21,28	21,17	21,02	21,25	21,21	21,05	21,69	21,12	21,05	21,05	20,93	21,02	21,05	21,09	21,19	21,21	21,12	21,05	34,8
	AUG	38,81	38,87	38,85	38,85	38,64	38,74	38,87	38,67	38,70	38,85	38,27	38,78	38,85	38,85	38,96	38,87	38,85	38,81	38,72	38,70	38,79	38,85	100
	AUI	40,10	40,10	40,10	40,10	40,08	40,09	40,10	40,08	40,09	40,10	40,05	40,09	40,10	40,10	40,11	40,10	40,10	40,10	40,09	40,09	40,09	40,10	92,1
Asp	CUA	29,52	29,47	29,51	29,52	29,55	29,43	29,43	29,45	29,28	29,52	29,55	29,52	29,56	29,52	29,52	29,62	29,52	29,51	29,41	29,52	29,46	29,48	38,5
	CUG	31,11	31,15	31,12	31,11	31,07	31,19	31,19	31,17	31,32	31,11	31,07	31,11	31,06	31,11	31,11	31,01	31,11	31,12	31,21	31,11	31,16	31,14	100
	CUI	39,38	39,38	39,38	39,38	39,37	39,38	39,38	39,38	39,40	39,38	39,37	39,38	39,37	39,38	39,38	39,37	39,38	39,38	39,39	39,38	39,38	39,38	94,2
Glu	CUU	67,83	67,83	67,92	67,83	67,83	67,83	67,83	67,22	67,83	67,83	67,05	67,83	67,83	67,83	67,83	67,83	67,83	67,71	67,83	67,83	66,72	67,58	100
	CUI	0,00	0,00	0,00	0,00	0,00	0,00	0,00	0,00	0,00	0,00	0,00	0,00	0,00	0,00	0,00	0,00	0,00	0,00	0,00	0,00	0,00	0,00	0
	CUC	32,17	32,17	32,07	32,17	32,17	32,17	32,17	32,77	32,17	32,17	32,94	32,17	32,17	32,17	32,17	32,17	32,17	32,29	32,17	32,17	33,27	32,41	84
Gln	GUU	62,22	62,10	62,10	62,10	62,16	62,10	62,10	61,72	62,10	62,10	62,10	62,03	62,16	62,10	62,16	62,10	62,03	62,16	62,10	62,22	62,28	63,17	75
	GUI	0,00	0,00	0,00	0,00	0,00	0,00	0,00	0,00	0,00	0,00	0,00	0,00	0,00	0,00	0,00	0,00	0,00	0,00	0,00	0,00	0,00	0,00	0
	GUC	37,77	37,90	37,90	37,90	37,84	37,90	37,90	38,28	37,90	37,90	37,90	37,96	37,84	37,90	37,84	37,90	37,96	37,84	37,90	37,77	37,71	36,83	100
Ala	CGA	19,68	19,79	19,79	19,80	19,79	20,25	20,48	19,79	19,77	19,76	20,54	19,76	19,72	19,87	19,79	19,74	19,83	19,82	19,65	19,79	19,65	19,86	34
	CGG	23,25	23,19	23,19	23,22	23,19	23,79	23,98	23,49	23,23	23,19	24,08	23,23	23,22	23,19	23,19	23,16	23,16	23,22	23,42	23,29	23,44	23,21	100
	CGI	26,23	26,24	26,24	26,26	26,24	26,89	27,14	26,46	26,26	26,22	27,24	26,25	26,23	26,28	26,24	26,19	26,24	26,27	26,34	26,31	26,36	26,28	88
	CGU	26,94	26,88	26,88	26,91	26,88	26,84	26,94	26,76	26,85	26,86	27,01	26,88	26,94	26,77	26,88	26,93	26,88	26,88	26,58	26,64	26,69	26,91	70
	CGC	3,90	3,89	3,89	3,82	3,89	2,23	1,45	3,50	3,88	3,97	3,59	3,89	3,90	3,89	3,89	3,97	3,89	3,81	4,01	3,96	3,86	3,74	25
Arg	UCU	77,11	77,11	77,11	77,11	77,11	77,11	77,11	77,11	77,11	77,11	77,11	77,11	76,91	77,11	73,77	77,11	76,97	77,11	77,11	76,94	76,38	77,20	100
	UCI	0,00	0,00	0,00	0,00	0,00	0,00	0,00	0,00	0,00	0,00	0,00	0,00	0,00	0,00	0,00	0,00	0,00	0,00	0,00	0,00	0,00	0,00	0
	UCC	22,88	22,88	22,88	22,88	22,88	22,88	22,88	22,88	22,88	22,88	22,88	22,88	22,73	22,88	26,22	22,88	22,84	22,88	22,88	23,05	23,61	22,80	50
	GCG	0,00	0,00	0,00	0,00	0,00	0,00	0,00	0,00	0,00	0,00	0,00	0,00	0,04	0,00	0,00	0,00	0,00	0,00	0,00	0,00	0,00	0,00	54
	GCI	0,00	0,00	0,00	0,00	0,00	0,00	0,00	0,00	0,00	0,00	0,00	0,00	0,07	0,00	0,00	0,00	0,00	0,00	0,00	0,00	0,00	0,00	47
	GCA	0,00	0,00	0,00	0,00	0,00	0,00	0,00	0,00	0,00	0,00	0,00	0,00	0,07	0,00	0,00	0,00	0,00	0,00	0,00	0,00	0,00	0,00	13
	GCU	0,00	0,00	0,00	0,00	0,00	0,00	0,00	0,00	0,00	0,00	0,00	0,00	0,04	0,00	0,00	0,00	0,18	0,00	0,00	0,00	0,00	0,00	57
	GCC	0,00	0,00	0,00	0,00	0,00	0,00	0,00	0,00	0,00	0,00	0,00	0,00	0,14	0,00	0,00	0,00	0,00	0,00	0,00	0,00	0,00	0,00	56

D) Anticodon variation		0.0 µg/ml AMD											0.05 µg/ml AMD									0.2 µg/ml AMD		
		P4	P5	P20	P36	P38	P41	P44	P65	P85	P103	P21/R	P4	P5	P20	P36	P38	P41	P44	P65	P85	P20	P38	Cell
Val	CAU	0,00	1,17	0,27	0,51	-1,39	1,92	0,00	0,71	-1,76	-0,15	-4,75	1,30	0,90	0,10	-2,62	-7,68	-7,02	-8,11	-6,94	-8,39	-10,33	-21,39	74
	CAC	0,00	1,17	2,21	0,51	-1,39	1,43	0,00	-0,26	-1,76	-0,64	-4,29	1,30	0,90	-0,38	-2,62	-7,68	-7,02	-8,11	-7,85	-7,95	-9,01	-10,02	100
	CAG	0,00	-0,81	-0,53	-0,32	0,69	-1,63	-0,10	0,05	1,25	-0,22	4,17	-0,76	-0,59	0,19	2,01	6,69	6,24	6,67	6,34	6,65	8,61	12,79	57
	CAI	0,00	-0,82	0,20	-0,14	-0,74	-3,87	-0,65	1,70	1,45	-2,58	9,47	-0,03	-0,41	0,92	3,11	14,04	13,64	12,30	13,38	11,76	18,14	17,02	29
	CAA	0,00	-0,80	-1,09	-0,46	1,80	0,11	0,32	-1,22	1,10	1,62	0,05	-1,33	-0,73	-0,38	1,16	0,98	0,50	2,29	0,88	2,69	1,21	9,49	28
Ile	UAI	0,00	0,20	-0,02	0,07	-0,34	0,03	-0,17	-0,09	-0,66	-0,20	-0,91	0,15	0,14	-0,06	-0,35	-1,50	-1,37	-1,63	-1,48	-2,41	-2,62	-2,01	91
	UAG	0,00	0,98	-0,35	0,33	-0,39	-0,19	-0,46	-0,68	-3,60	0,09	-3,75	0,47	0,47	-0,16	-1,85	-6,35	-5,78	-6,74	-6,35	-10,81	-9,59	-8,13	100
	UAA	0,00	-0,60	0,31	-0,20	-0,28	0,26	0,12	0,51	2,36	-0,50	2,00	-0,17	-0,21	0,05	1,19	3,47	3,14	3,60	3,51	6,20	4,52	4,25	33
	UAU	0,00	-1,06	0,00	-0,36	2,47	-0,36	1,12	0,36	3,33	1,61	5,27	-0,94	-0,83	0,36	1,82	8,54	7,84	9,39	8,40	13,44	15,72	11,72	33
Ser	AGA	0,00	-0,65	-0,08	0,16	0,00	-0,39	-0,08	0,08	-0,53	-0,24	-0,95	-0,57	-1,09	1,40	1,49	1,41	3,20	3,95	2,21	3,04	3,10	-1,14	38
	AGG	0,00	-0,65	-0,08	0,16	0,00	-0,08	0,19	0,08	-0,19	-0,24	-0,95	-0,57	-0,75	1,40	0,95	-1,57	-0,73	0,21	-1,28	-0,42	-0,35	-1,48	100
	AGI	0,00	-0,65	-0,08	0,16	0,00	-0,23	0,05	0,08	-0,36	-0,24	-0,95	-0,57	-0,92	1,40	1,22	-0,09	-0,43	0,19	-1,39	-0,46	-0,50	-1,31	94
	AGU	0,00	-1,15	-0,37	-0,12	-0,57	-0,46	-0,37	-0,20	-0,18	-0,53	-1,23	-1,07	-0,89	1,12	1,20	1,19	-1,76	-1,40	-0,24	-0,21	-1,22	-1,70	77
	UCA	0,00	-0,65	-0,08	0,16	2,00	-0,24	-0,08	0,08	-0,24	-0,24	-6,33	-0,57	-0,81	1,40	1,49	1,41	1,58	0,31	-0,66	1,79	-3,99	-4,87	27
	UCG	0,00	18,95	4,30	-2,03	3,05	6,32	2,11	0,08	6,32	8,50	-6,73	16,87	18,76	-34,16	-31,88	-11,93	-1,73	-15,08	1,52	-12,48	-6,73	-6,73	97
	UCI	0,00	11,76	2,69	-1,23	2,67	3,91	1,31	0,08	3,91	5,29	-1,150	10,47	11,58	-21,11	-19,64	-7,04	-0,52	-9,43	0,72	-7,24	-1,15	-1,15	86
	AGC																							23
Leu	AAC	0,00	0,17	0,00	0,26	0,17	0,18	0,01	0,59	-0,12	-0,96	0,11	-0,18	-0,11	0,26	0,43	0,17	0,17	-0,38	0,80	-1,22	0,17	0,09	26
	AAU	0,00	0,42	0,00	0,17	0,08	0,18	0,10	0,34	0,22	0,42	0,19	0,16	-0,19	0,17	-0,08	0,08	1,44	1,56	0,21	0,59	0,08	0,43	24
	AAI																							0
	GAA	0,00	-0,66	-0,66	-0,57	-0,66	-0,48	-0,48	-0,57	0,55	1,08	-0,39	0,48	1,05	-0,57	-0,57	-1,32	-0,66	-0,54	-1,02	0,99	0,00	-0,57	13
	GAG	0,00	-0,66	-0,66	-0,57	-0,66	-0,48	-0,48	-0,57	0,55	1,08	-0,39	0,48	1,05	-0,57	-0,57	-1,32	-0,66	-0,54	-1,02	0,99	0,00	-0,57	44
	GAI	0,00	-0,66	-0,66	-0,57	-0,66	-0,48	-0,48	-0,57	0,55	1,08	-0,39	0,48	1,05	-0,57	-0,57	-1,32	-0,66	-0,54	-1,02	0,99	0,00	-0,57	39
	GAC	0,00	-0,50	0,00	-0,41	0,00	-0,32	0,18	-1,40	-0,45	0,74	-0,23	-0,18	-0,77	-0,41	-0,41	0,50	-1,99	-1,53	-4,82	0,65	-0,50	-0,41	100
	GAU	0,00	-0,50	2,06	-0,41	0,00	-0,32	0,18	-1,40	-0,45	0,74	-0,23	-0,18	1,28	-0,41	-0,41	0,50	-1,99	0,51	9,51	2,74	-0,50	-0,41	52
Gly	CCU	0,00	-0,03	-0,03	0,00	0,39	-1,00	-1,43	0,51	-0,07	-0,14	-1,75	-0,03	-0,14	-0,03	-0,03	0,09	-0,03	-0,03	1,39	0,02	0,09	-0,27	100
	CCI	0,00	0,34	0,37	0,00	-0,01	-2,16	-3,27	0,16	0,25	0,50	-3,67	0,37	0,50	0,37	0,37	0,21	0,34	0,37	-0,44	0,48	0,19	0,47	71
	CCA	0,00	0,69	0,40	0,00	-0,05	-2,13	-3,24	-1,95	0,29	0,57	-3,89	0,40	0,57	0,40	0,40	0,52	0,69	0,40	-3,33	0,52	0,81	0,47	20
	CCG	0,00	0,00	0,33	0,00	0,02	-2,20	-3,31	2,24	0,21	0,42	-3,45	0,33	0,42	0,33	0,33	-0,09	0,00	0,33	2,42	0,45	-0,42	0,47	82
	CCC	0,00	-1,20	-1,20	0,00	-1,08	10,43	15,53	-1,55	-0,66	-1,32	17,90	-1,20	-1,32	-1,20	-1,20	-1,08	-1,20	-1,20	-1,86	-1,74	-1,08	-0,85	62
Asn	UUA	0,00	-0,15	-0,15	-0,15	-0,40	-0,44	-0,32	-0,26	0,25	-0,15	-0,15	-0,15	-0,15	-0,15	0,00	-0,15	-0,15	-0,13	0,37	-0,13	0,31	-0,26	36
	UUG	0,00	0,13	0,13	0,13	0,37	0,40	0,29	0,23	-0,23	0,13	0,13	0,13	0,13	0,13	0,00	0,13	0,13	0,12	-0,34	0,12	-0,29	0,23	100
	UUI	0,00	0,02	0,02	0,02	0,05	0,05	0,04	0,03	-0,03	0,02	0,02	0,02	0,02	0,02	0,00	0,02	0,02	0,02	-0,04	0,02	-0,04	0,03	89
Cys	ACA	0,00	0,00	-0,17	-0,17	-0,17	-0,17	0,00	-0,17	-0,66	0,00	-0,17	-0,17	-0,17	0,00	-0,17	0,00	-0,17	-0,17	0,33	-0,17	-0,17	-0,17	36
	ACG	0,00	0,00	0,14	0,14	0,14	0,14	0,00	0,14	0,56	0,00	0,14	0,14	0,14	0,00	0,14	0,00	0,14	0,14	-0,27	0,14	0,14	0,14	100
	ACI	0,00	0,00	0,01	0,01	0,01	0,01	0,00	0,01	0,04	0,00	0,01	0,01	0,01	0,00	0,01	0,00	0,01	0,01	-0,02	0,01	0,01	0,01	93
Thr	UGA	0,00	0,14	-0,04	-0,16	-0,26	0,06	-0,18	-0,21	0,08	2,06	-0,77	0,10	0,12	-0,62	-0,58	0,26	0,41	0,40	0,91	0,04	0,88	0,75	30
	UGG	0,00	-2,04	-0,32	0,40	0,36	-0,22	-0,06	-1,33	-1,04	0,41	7,95	-1,80	-2,56	3,75	3,52	-3,04	-4,31	-2,96	-5,29	-0,85	-5,60	-5,72	100
	UGI	0,00	0,32	0,05	-0,07	-0,09	0,06	-0,01	0,15	0,17	0,38	-1,20	0,28	0,38	-0,61	-0,57	0,35	0,67	0,49	0,91	0,14	0,88	0,93	67
	UGU	0,00	0,45	0,10	-0,02	0,05	0,01	0,11	0,47	0,22	-1,35	-1,68	0,41	0,60	-0,65	-0,61	0,47	0,90	0,54	0,86	0,19	0,88	1,07	99
	UGC																							31

D) Anticodon variation		0.0 µg/ml AMD											0.05 µg/ml AMD									0.2 µg/ml AMD		
		P4	P5	P20	P36	P38	P41	P44	P65	P85	P103	P21/R	P4	P5	P20	P36	P38	P41	P44	P65	P85	P20	P38	Cell
Pro	GGA	0,00	-0,05	0,09	-0,14	-0,18	0,51	-0,38	-0,09	-0,49	-0,09	-0,86	0,00	0,23	-0,29	0,06	0,19	-0,23	0,09	0,68	-0,38	-0,09	0,04	37
	GGG	0,00	-0,05	0,09	-0,14	-0,18	0,41	-0,28	-0,09	-0,29	-0,09	-0,96	0,59	0,23	-0,19	-0,04	0,09	-0,23	0,09	0,68	-0,28	-0,09	0,04	94
	GGI	0,00	-0,05	0,09	-0,14	-0,18	0,46	-0,33	-0,09	-0,39	-0,09	-0,91	0,30	0,23	-0,24	0,00	0,14	-0,23	0,09	0,68	-0,33	-0,09	0,04	89
	GGU	0,00	0,52	0,38	0,49	0,66	0,47	1,15	0,68	0,96	0,68	4,30	0,43	-0,83	0,82	0,71	0,57	0,47	0,38	0,00	0,79	0,68	0,54	100
	GGC	0,00	-7,32	-7,19	-5,59	-7,44	-14,39	-12,77	-9,18	-8,93	-9,18	-5,33	-10,91	9,34	-9,05	-11,16	-11,03	-3,86	-7,19	-10,30	-7,32	-9,18	-9,05	17
Lys	UUU	0,00	0,00	0,00	0,00	0,00	0,00	0,00	-0,31	0,00	0,00	-0,10	0,00	-0,31	0,00	0,00	0,00	0,00	0,00	0,57	-0,10	1,60	0,08	64
	UUC	0,00	0,00	0,00	0,00	0,00	0,00	0,00	0,55	0,00	0,00	0,18	0,00	0,55	0,00	0,00	0,00	0,00	0,00	-1,00	0,18	-2,80	-0,14	100
	UUI																							0
His	GUA	0,00	0,50	0,50	0,50	-1,48	-0,99	0,50	-1,95	-2,90	0,50	-6,94	0,00	0,50	0,50	-1,95	0,50	0,50	0,00	0,00	0,00	0,50	-2,01	34
	GUG	0,00	-0,76	-0,76	-0,76	2,26	1,51	-0,76	2,98	4,43	-0,76	10,62	0,00	-0,76	-0,76	2,98	-0,76	-0,76	0,00	-0,01	-0,01	-0,76	3,08	100
	GUI	0,00	-0,04	-0,04	-0,04	0,13	0,08	-0,04	0,17	0,25	-0,04	0,59	0,00	-0,04	-0,04	0,17	-0,04	-0,04	0,00	0,00	0,00	-0,04	0,17	92
Phe	AAA	0,00	-0,21	-0,21	-0,19	0,11	-0,11	-0,08	-0,21	-1,08	-1,43	-0,27	-0,11	-0,23	-0,21	0,11	-0,23	-0,32	-0,08	-0,29	-1,36	-0,11	-0,13	36
	AAG	0,00	0,20	0,20	0,18	-0,10	0,10	0,08	0,20	1,00	1,33	0,25	0,10	0,22	0,20	-0,10	0,22	0,29	0,08	0,27	1,26	0,10	0,12	100
	AAI	0,00	0,01	0,01	0,01	-0,01	0,01	0,01	0,01	0,07	0,10	0,02	0,01	0,02	0,01	-0,01	0,02	0,02	0,01	0,02	0,09	0,01	0,01	93
Tyr	AUA	0,00	-0,33	-0,18	-0,18	0,88	0,37	-0,33	0,73	0,55	-0,18	2,81	0,15	-0,18	-0,18	-0,79	-0,33	-0,18	0,00	0,45	0,55	0,12	-0,18	34,8
	AUG	0,00	0,17	0,09	0,09	-0,44	-0,18	0,17	-0,36	-0,27	0,09	-1,40	-0,08	0,09	0,09	0,39	0,17	0,09	0,00	-0,22	-0,27	-0,06	0,09	100,0
	AUI	0,00	0,01	0,01	0,01	-0,04	-0,02	0,01	-0,03	-0,02	0,01	-0,13	-0,01	0,01	0,01	0,04	0,01	0,01	0,00	-0,02	-0,02	-0,01	0,01	92,1
Asp	CUA	0,00	-0,15	-0,04	0,00	0,12	-0,31	-0,30	-0,23	-0,79	0,00	0,12	0,00	0,15	0,00	0,00	0,34	0,00	-0,04	-0,37	0,00	-0,19	-0,12	38,5
	CUG	0,00	0,13	0,03	0,00	-0,10	0,27	0,26	0,20	0,69	0,00	-0,10	0,00	-0,13	0,00	0,00	-0,30	0,00	0,03	0,32	0,00	0,17	0,10	100,0
	CUI	0,00	0,01	0,00	0,00	-0,01	0,02	0,02	0,01	0,05	0,00	-0,01	0,00	-0,01	0,00	0,00	-0,02	0,00	0,00	0,02	0,00	0,01	0,01	94,2
Glu	CUU	0,00	0,00	0,14	0,00	0,00	0,00	0,00	-0,89	0,00	0,00	-1,15	0,00	0,00	0,00	0,00	0,00	0,00	-0,18	0,00	0,00	-1,63	-0,36	100
	CUI																							0
	CUC	0,00	0,00	-0,29	0,00	0,00	0,00	0,00	1,89	0,00	0,00	2,42	0,00	0,00	0,00	0,00	0,00	0,00	0,38	0,00	0,00	3,44	0,76	84
Gln	GUU	0,00	-0,20	-0,20	-0,20	-0,10	-0,20	-0,20	-0,81	-0,20	-0,20	-0,20	-0,30	-0,10	-0,20	-0,10	-0,20	-0,30	-0,10	-0,20	0,00	0,10	1,52	75
	GUI																							0
	GUC	0,00	0,33	0,33	0,33	0,17	0,33	0,33	1,34	0,33	0,33	0,33	0,50	0,17	0,33	0,17	0,33	0,50	0,17	0,33	0,00	-0,17	-2,50	100
Ala	CGA	0,00	0,58	0,58	0,59	0,58	2,90	4,08	0,58	0,48	0,39	4,34	0,38	0,20	0,97	0,58	0,29	0,77	0,68	-0,15	0,57	-0,14	0,89	34
	CGG	0,00	-0,25	-0,25	-0,16	-0,25	2,32	3,15	1,03	-0,09	-0,28	3,57	-0,11	-0,14	-0,27	-0,25	-0,38	-0,39	-0,15	0,73	0,18	0,82	-0,20	100
	CGI	0,00	0,05	0,05	0,11	0,05	2,53	3,49	0,87	0,11	-0,04	3,85	0,07	-0,02	0,18	0,05	-0,14	0,03	0,15	0,42	0,32	0,48	0,19	88
	CGU	0,00	-0,23	-0,23	-0,11	-0,23	-0,39	0,00	-0,69	-0,33	-0,30	0,27	-0,22	-0,01	-0,62	-0,23	-0,02	-0,23	-0,22	-1,34	-1,10	-0,94	-0,10	70
	CGC	0,00	-0,23	-0,23	-2,02	-0,23	-42,84	-62,90	-10,20	-0,33	1,99	-7,96	-0,22	-0,01	-0,24	-0,23	1,88	-0,23	-2,12	2,81	1,56	-0,94	-3,92	25
Arg	UCU	0,00	0,00	0,00	0,00	0,00	0,00	0,00	0,00	0,00	0,00	0,00	0,00	-0,27	0,00	-4,33	0,00	-0,18	0,00	0,00	-0,22	-0,95	0,11	100
	UCI																							0
	UCC	0,00	0,00	0,00	0,00	0,00	0,00	0,00	0,00	0,00	0,00	0,00	0,00	-0,68	0,00	14,59	0,00	-0,18	0,00	0,00	0,73	3,19	2,03	50
	GCG																							54
	GCI																							47
	GCA																							13
	GCU																							57
	GCC																							56

Those anticodons whose initial counts were 0 were omitted in the analysis of the anticodon usage variation.

Those anticodons whose variation was exceptional (only in a few pasages) and inconsistent were omitted in the analysis of the anticodon usage variation.

Anticodons Ser UCG and UCI were also ommitted from the analysis since they are associated with a single position (VP1 197) with continuous variability, independent of AMD [30].
