# Supplementary material for: Fine-Tuning Translation Kinetics Selection as the Driving Force of Codon Usage Bias in the Hepatitis A Virus Capsid
Source: PLoS Pathog. 2010 Mar 5;6(3):e1000797. doi: 10.1371/journal.ppat.1000797 (PMC2832697; doi:10.1371/journal.ppat.1000797)
Supplement: Table S5 — Conversion from codon usage tables to anticodon usage variation in the polymerase region studied. A) Codon counts in 50 molecular clones of the different populations studied. B) Anticodon usage estimation from the codon counts, applying the multiple codon:anticodon pairing and the codon:anticodon coupling efficiencies described elsewhere [19]. C) Usage of each anticodon (in percentage) out of the total usage of the anticodons bearing the same aminoacid. D) Variation (increasing or decreaing) of the relative usage of each anticodon from the initial passage. (0.03 MB PDF) [file ppat.1000797.s005.pdf]

Table S5. Conversion from codon usage tables to anticodon usage variation in the polymerase region studied. A) Codon counts in 50 molecular clones of the different populations studied. B) Anticodon usage estimation from the codon counts, applying the multiple codon:anticodon pairing and the codon:anticodon coupling efficiencies described in (dos Reis M, Savva R. Wernisch L (2004) Solving the riddle of codon usage preferences: a test for tranlational selection. Nucl Acids Res 32:5036-5044) C) Usage of each anticodon (in percentage) out of the total usage of the anticodons bearing the same aminoacid. D) Variation (increasing or decreaing) of the relative usage of each anticodon from the initial passage.

**A) Codon usage**		0.0 µg/ml AMD											0.05 µg/ml AMD									0.2 µg/ml AMD		
		P4	P5	P20	P36	P38	P41	P44	P65	P85	P103	P21/R	P4	P5	P20	P36	P38	P41	P44	P65	P85	P20	P38	Cell
Val	GUU	302	301	324	316	329	314	310	345	308	300	326	317	323	309	334	341	339	340	331	338	348	350	34
	GUG	250	250	250	250	250	250	250	250	250	250	250	250	250	250	250	250	249	250	251	250	250	250	100
	GUA	50	50	50	50	50	50	50	50	50	50	50	51	50	50	50	50	50	50	51	50	50	50	19
	GUC	149	149	147	150	150	150	150	150	150	150	150	150	148	150	150	151	150	150	148	150	150	150	53
Ile	AUU	750	750	750	749	750	748	748	750	750	750	750	751	751	753	750	750	751	750	747	750	752	750	61
	AUA	149	150	150	151	150	150	150	150	150	150	151	150	150	150	150	150	150	151	151	150	150	150	24
	AUC	150	150	150	150	149	152	152	150	150	150	150	149	149	147	150	150	149	150	153	150	148	150	100
Ser	UCU	250	249	250	250	250	250	250	250	250	250	249	250	250	250	250	250	250	251	250	250	250	249	71
	UCA	250	251	257	254	250	255	255	256	281	250	287	250	250	250	250	252	257	256	257	396	292	250	50
	AGU	200	200	200	200	200	200	200	200	199	199	200	200	200	199	200	199	200	199	200	199	200	200	50
	UCC	50	50	50	50	50	50	50	48	48	50	50	50	50	50	50	50	50	50	50	50	50	50	95
	UCG	0	0	0	0	0	0	0	2	2	0	0	0	0	0	0	2	0	0	1	0	0	0	22
	AGC	0	0	0	0	0	0	0	0	1	0	0	0	0	1	1	1	0	1	0	1	0	0	100
Leu	UUG	450	449	450	449	451	449	449	450	412	450	450	450	450	450	448	448	450	448	449	450	448	450	26
	UUA	400	400	396	396	395	398	402	404	370	400	369	400	400	401	408	424	417	414	420	360	361	400	12
	CUU	200	199	200	200	201	199	200	200	200	200	200	199	200	200	200	200	200	201	200	201	200	154	25
	CUG	100	101	100	100	100	100	100	100	100	100	101	100	100	100	101	100	99	99	100	100	102	100	100
	CUA	100	100	97	101	101	98	94	90	98	100	94	100	100	99	92	74	74	81	73	86	97	100	15
	CUC	0	0	0	0	0	0	0	0	0	0	0	0	0	0	0	0	0	0	0	0	0	46	47
Gly	GGA	200	200	200	200	199	200	201	200	204	200	226	200	200	200	200	200	200	200	200	233	239	246	67
	GGU	399	400	350	386	370	385	382	356	348	396	364	399	399	387	362	356	367	357	368	348	350	350	44
	GGG	50	50	50	50	50	50	50	50	46	50	24	50	50	50	50	50	50	50	48	18	12	4	68
	GGC	51	50	69	64	80	63	58	94	102	54	86	51	51	63	88	92	83	90	84	102	99	100	100
Asn	AAU	350	350	351	351	350	352	355	350	350	350	350	349	349	349	351	350	350	354	350	349	349	350	73
	AAC	50	50	49	49	50	50	50	50	50	50	50	52	51	51	49	51	50	50	50	50	50	50	100
Cys	UGU	200	201	200	200	200	200	200	200	200	200	201	200	200	200	200	200	200	200	200	200	200	200	68
	UGC	50	49	50	50	50	50	50	50	50	49	50	50	50	50	50	50	50	49	50	51	50	50	100
Thr	ACU	151	150	150	150	151	151	150	150	149	150	152	150	150	151	150	150	151	150	150	150	150	152	55
	ACA	300	300	299	300	301	300	300	300	301	300	299	300	300	300	300	300	300	301	300	300	300	300	63
	ACC	149	150	150	150	148	149	150	150	150	151	148	150	150	149	150	149	149	150	150	150	150	149	100
	ACG	50	50	51	50	50	50	50	50	50	49	50	50	50	50	50	50	50	50	50	50	50	50	29
Pro	CCU	300	300	303	304	307	302	299	334	312	301	325	301	300	302	301	311	321	321	321	337	346	350	78
	CCA	450	450	450	450	450	450	451	450	450	450	450	450	450	450	450	450	450	450	450	450	450	450	73
	CCC	100	100	97	96	93	98	100	66	88	100	75	100	100	98	91	89	79	79	79	62	54	50	100
	CCG	0	0	0	0	0	0	0	0	0	0	0	0	0	0	0	0	0	0	0	0	0	0	33
Lys	AAA	600	600	600	600	600	599	600	600	600	600	601	600	600	601	600	600	600	597	597	601	601	600	64
	AAG	200	200	200	200	200	200	200	200	200	200	199	200	200	199	200	200	200	200	201	200	201	200	100
His	CAU	200	200	201	200	200	200	200	202	200	199	200	200	200	200	200	200	198	196	200	200	200	200	65
	CAC	50	51	49	50	50	50	50	50	50	50	50	50	50	50	51	50	50	52	50	50	50	50	100

**A) Codon usage**		0.0 µg/ml AMD											0.05 µg/ml AMD									0.2 µg/ml AMD		
		P4	P5	P20	P36	P38	P41	P44	P65	P85	P103	P21/R	P4	P5	P20	P36	P38	P41	P44	P65	P85	P20	P38	Cell
Phe	UUU	599	599	600	600	599	605	604	600	600	600	600	599	600	601	600	600	600	596	602	600	599	600	70
	UUC	201	201	200	200	204	196	196	200	200	200	200	201	200	199	200	200	200	204	200	200	201	200	100
Tyr	UAU	350	351	350	350	350	350	351	348	350	349	350	350	350	350	351	350	352	350	348	350	350	350	66
	UAC	50	49	50	50	50	50	49	50	50	52	50	50	50	50	49	50	50	49	52	50	50	50	100
Asp	GAU	1100	1100	1099	1099	1100	1100	1100	1100	1100	1098	1099	1100	1100	1100	1099	1102	1100	1099	1099	1100	1100	1100	75
	GAC	0	0	1	1	0	0	0	0	0	8	1	0	0	0	1	1	0	2	1	0	0	0	100
Glu	GAA	450	450	450	450	450	451	450	450	450	450	450	449	451	450	450	449	450	449	449	450	450	450	65
	GAG	250	250	250	250	250	250	250	250	250	250	250	251	249	250	250	249	251	249	250	250	250	250	100
Gln	CAA	50	50	50	50	51	50	50	50	50	50	50	50	50	50	50	50	51	50	50	52	50	50	33
	CAG	250	250	250	250	249	250	250	250	250	250	250	250	250	250	249	250	249	250	250	248	250	250	100
Ala	GCU	599	600	579	585	571	586	592	555	595	600	574	582	579	558	564	559	561	560	567	568	552	550	67
	GCA	100	100	100	99	101	100	100	100	97	100	100	99	100	100	100	100	100	99	100	94	100	100	48
	GCC	100	100	100	100	100	100	98	100	100	100	100	100	100	100	100	100	100	102	102	100	100	100	100
	GCG	0	0	0	0	0	0	0	0	0	0	0	1	0	0	0	0	0	0	0	0	0	0	25
Arg	AGA	501	499	500	500	500	500	500	500	500	501	500	500	500	500	500	500	501	503	500	499	499	500	88
	AGG	0	0	0	0	0	0	0	0	0	0	0	0	0	0	0	0	0	0	0	0	0	0	98
	CGC	0	0	0	0	0	0	0	0	0	0	0	0	0	0	0	0	0	0	0	0	0	0	100
	CGU	0	0	0	0	0	0	0	0	0	0	0	0	0	0	0	0	0	0	0	0	0	0	42
	CGA	50	51	49	50	50	50	50	50	50	50	50	50	50	50	50	50	49	50	50	51	50	50	48
	CGG	0	0	1	0	0	0	0	0	0	0	0	0	0	0	0	0	1	0	0	0	0	0	92

**B) Anticodon usage**		0.0 µg/ml AMD											0.05 µg/ml AMD									0.2 µg/ml AMD		
		P4	P5	P20	P36	P38	P41	P44	P65	P85	P103	P21/R	P4	P5	P20	P36	P38	P41	P44	P65	P85	P20	P38	Cell
Val	CAC	190	190	190	190	190	190	190	190	190	190	190	190	190	190	190	190	189	190	190	190	190	190	76
	CAU	143	143	143	143	143	143	143	143	143	143	143	144	143	143	143	143	142	143	144	143	143	143	56
	CAG	199	198	206	204	209	204	202	215	201	198	208	205	206	202	211	214	213	213	209	213	216	217	43
	CAI	62	62	62	63	63	63	63	63	63	63	63	63	62	63	63	63	63	63	62	63	63	63	22
	CAA	190	189	204	199	207	198	195	217	194	189	205	199	203	194	210	214	213	214	208	213	219	220	21
Ile	UAG	258	258	258	258	258	259	259	258	258	258	258	258	258	257	258	258	258	258	259	258	257	258	72
	UAI	352	352	352	352	352	352	352	352	352	352	352	352	352	352	352	352	352	352	352	352	352	352	65
	UAA	290	290	290	289	290	289	289	290	290	290	290	290	290	291	290	290	290	290	288	290	290	290	24
	UAU	149	150	150	151	150	150	150	150	150	150	151	150	150	150	150	150	150	151	151	150	150	150	24
Ser	AGA	97	96	97	97	97	97	97	97	97	97	96	97	97	97	97	97	97	97	97	97	97	96	27
	AGG	86	86	86	86	86	86	86	85	85	86	86	86	86	86	86	86	86	86	86	86	86	86	71
	AGI	117	117	117	117	117	117	117	117	117	117	117	117	117	117	117	117	117	118	117	117	117	117	67
	AGU	250	251	257	254	250	255	255	256	281	250	287	250	250	250	250	252	257	256	257	396	292	250	55
	UCA	77	77	77	77	77	77	77	77	77	77	77	77	77	77	77	77	77	77	77	77	77	77	19
	UCG	46	46	46	46	46	46	46	46	46	45	46	46	46	46	46	46	46	46	46	46	46	46	70
	UCI	77	77	77	77	77	77	77	77	77	77	77	77	77	77	78	77	77	77	77	77	77	77	61
	AGC	0	0	0	0	0	0	0	2	2	0	0	0	0	0	0	2	0	0	1	0	0	0	17
Leu	AAC	341	340	341	340	342	340	340	341	312	341	341	341	341	341	340	340	341	340	340	341	340	341	20
	AAU	509	509	505	505	504	507	511	513	470	509	478	509	509	510	516	532	526	522	529	469	469	509	18
	AAI	0	0	0	0	0	0	0	0	0	0	0	0	0	0	0	0	0	0	0	0	0	0	0
	GAA	77	77	77	77	78	77	77	77	77	77	77	77	77	77	77	77	77	78	77	78	77	59	10
	GAG	45	45	45	45	46	45	45	45	45	45	45	45	45	45	45	45	45	46	45	46	45	62	33
	GAI	77	77	77	77	78	77	77	77	77	77	77	77	77	77	77	77	77	78	77	78	77	79	29
	GAC	76	77	76	76	76	76	76	76	76	76	77	76	76	76	77	76	75	75	76	76	77	76	76
	GAU	124	124	121	125	125	122	118	114	122	124	118	124	124	123	116	98	98	105	97	110	122	124	39
Gly	CCU	212	212	212	212	211	212	213	212	215	212	232	212	212	212	212	212	212	212	212	237	242	247	83
	CCI	175	175	164	176	176	175	172	177	177	176	177	175	175	176	177	176	176	176	177	177	177	177	59
	CCA	154	154	135	149	143	149	147	137	134	153	141	154	154	149	140	137	142	138	142	134	135	135	17
	CCG	121	120	120	125	131	124	121	136	139	122	133	121	121	125	134	135	132	134	133	139	137	138	68
	CCC	38	38	38	38	38	38	38	38	35	38	18	38	38	38	38	38	38	38	36	14	9	3	51
Asn	UUA	135	135	135	135	135	136	137	135	135	135	135	135	135	135	135	135	135	137	135	135	135	135	28
	UUG	126	126	126	126	126	127	127	126	126	126	126	127	126	126	126	127	126	127	126	126	126	126	78
	UUI	156	156	156	156	156	157	158	156	156	156	156	157	156	156	156	156	156	158	156	156	156	156	70
Cys	ACA	77	78	77	77	77	77	77	77	77	77	78	77	77	77	77	77	77	77	77	77	77	77	26
	ACG	75	74	75	75	75	75	75	75	75	74	75	75	75	75	75	75	75	74	75	75	75	75	74
	ACI	98	98	98	98	98	98	98	98	98	98	99	98	98	98	98	98	98	98	98	99	98	98	68
Thr	UGA	58	58	58	58	58	58	58	58	58	58	59	58	58	58	58	58	58	58	58	58	58	59	21
	UGG	121	121	121	121	120	121	121	121	121	122	121	121	121	121	121	121	121	121	121	121	121	121	71
	UGI	184	184	183	184	184	184	184	184	184	184	184	184	184	184	184	184	184	184	184	184	184	184	48
	UGU	312	312	311	312	313	312	312	312	313	312	311	312	312	312	312	312	312	313	312	312	312	312	70
	UGC	38	38	39	38	38	38	38	38	38	37	38	38	38	38	38	38	38	38	38	38	38	38	22

**B) Anticodon usage**		0.0 µg/ml AMD											0.05 µg/ml AMD									0.2 µg/ml AMD		
		P4	P5	P20	P36	P38	P41	P44	P65	P85	P103	P21/R	P4	P5	P20	P36	P38	P41	P44	P65	P85	P20	P38	Cell
Pro	GGA	116	116	117	117	119	117	115	129	120	116	125	116	116	117	116	120	124	124	124	130	134	135	30
	GGG	126	126	125	125	124	125	126	114	122	126	117	126	126	125	121	122	119	119	119	113	110	109	76
	GGI	158	158	158	158	158	158	157	157	157	158	157	158	158	158	154	157	157	157	157	156	156	156	72
	GGU	450	450	450	450	450	450	451	450	450	450	450	450	450	450	450	450	450	450	450	450	450	450	81
	GGC	0	0	0	0	0	0	0	0	0	0	0	0	0	0	0	0	0	0	0	0	0	0	14
Lys	UUU	600	600	600	600	600	599	600	600	600	600	601	600	600	601	600	600	600	597	597	601	601	600	64
	UUC	200	200	200	200	200	200	200	200	200	200	199	200	200	199	200	200	200	200	201	200	201	200	100
	UUI	0	0	0	0	0	0	0	0	0	0	0	0	0	0	0	0	0	0	0	0	0	0	0
His	GUA	77	77	78	77	77	77	77	78	77	77	77	77	77	77	77	77	76	76	77	77	77	77	25
	GUG	75	75	74	75	75	75	75	75	75	74	75	75	75	75	75	75	74	75	75	75	75	75	73
	GUI	98	99	98	98	98	98	98	99	98	98	98	98	98	98	99	98	97	97	98	98	98	98	67
Phe	AAA	231	231	232	232	231	234	233	232	232	232	232	231	232	232	232	232	232	230	232	232	231	232	27
	AAG	253	253	253	253	255	252	252	253	253	253	253	253	253	253	253	253	253	254	253	253	253	253	74
	AAI	315	315	315	315	317	316	315	315	315	315	315	315	315	315	315	315	315	316	316	315	315	315	69
Tyr	AUA	135	135	135	135	135	135	135	134	135	135	135	135	135	135	135	135	136	135	134	135	135	135	25
	AUG	109	108	109	109	109	109	108	108	109	110	109	109	109	109	108	109	109	108	110	109	109	109	73
	AUI	156	156	156	156	156	156	156	155	156	157	156	156	156	156	156	156	157	156	156	156	156	156	67
Asp	CUA	425	425	424	424	425	425	425	425	425	424	424	425	425	425	424	425	425	424	424	425	425	425	29
	CUG	251	251	251	251	251	251	251	251	251	255	251	251	251	251	251	252	251	252	251	251	251	251	75
	CUI	425	425	425	425	425	425	425	425	425	427	425	425	425	425	425	426	425	425	425	425	425	425	71
Glu	CUU	510	510	510	510	510	511	510	510	510	510	510	510	511	510	510	509	511	509	509	510	510	510	89
	CUI	0	0	0	0	0	0	0	0	0	0	0	0	0	0	0	0	0	0	0	0	0	0	0
	CUC	187	187	187	187	187	187	187	187	187	187	187	187	186	187	187	186	187	186	187	187	187	187	75
Gln	GUU	110	110	110	110	111	110	110	110	110	110	110	110	110	110	110	110	111	110	110	112	110	110	57
	GUI	0	0	0	0	0	0	0	0	0	0	0	0	0	0	0	0	0	0	0	0	0	0	0
	GUC	190	190	190	190	189	190	190	190	190	190	190	190	190	190	189	190	189	190	190	188	190	190	76
Ala	CGA	231	232	223	226	220	226	229	214	230	232	222	225	223	215	218	216	217	216	219	219	213	212	26
	CGG	225	225	219	221	217	221	222	212	224	225	218	220	219	213	215	214	214	215	217	216	212	211	77
	CGI	273	274	265	268	262	268	270	256	272	274	263	267	265	257	260	258	258	259	262	261	255	254	68
	CGU	100	100	100	99	101	100	100	100	97	100	100	99	100	100	100	100	100	99	100	94	100	100	54
	CGC	0	0	0	0	0	0	0	0	0	0	0	1	0	0	0	0	0	0	0	0	0	0	19
Arg	UCU	501	499	500	500	500	500	500	500	500	501	500	500	500	500	500	500	501	503	500	499	499	500	124
	UCI	0	0	0	0	0	0	0	0	0	0	0	0	0	0	0	0	0	0	0	0	0	0	0
	UCC	0	0	0	0	0	0	0	0	0	0	0	0	0	0	0	0	0	0	0	0	0	0	62
	GCG	0	0	0	0	0	0	0	0	0	0	0	0	0	0	0	0	0	0	0	0	0	0	68
	GCI	0	0	0	0	0	0	0	0	0	0	0	0	0	0	0	0	0	0	0	0	0	0	58
	GCA	0	0	0	0	0	0	0	0	0	0	0	0	0	0	0	0	0	0	0	0	0	0	16
	GCU	50	51	49	50	50	50	50	50	50	50	50	50	50	50	50	50	49	50	50	51	50	50	70
	GCC	0	0	1	0	0	0	0	0	0	0	0	0	0	0	0	0	1	0	0	0	0	0	70

C) Relative anticodon usage		0.0 µg/ml AMD											0.05 µg/ml AMD									0.2 µg/ml AMD		
		P4	P5	P20	P36	P38	P41	P44	P65	P85	P103	P21/R	P4	P5	P20	P36	P38	P41	P44	P65	P85	P20	P38	Cell
Val	CAU	18,22	18,25	17,77	17,88	17,60	17,93	18,02	17,26	18,06	18,25	17,66	17,96	17,77	18,04	17,49	17,32	17,36	17,36	17,72	17,40	17,19	17,15	74
	CAC	24,19	24,22	23,59	23,74	23,36	23,80	23,92	22,91	23,98	24,22	23,45	23,68	23,59	23,95	23,22	22,99	23,01	23,05	23,39	23,10	22,82	22,77	100
	CAG	25,36	25,34	25,60	25,60	25,79	25,58	25,52	26,01	25,49	25,37	25,75	25,59	25,62	25,50	25,86	25,99	25,96	25,94	25,67	25,91	26,05	26,07	57
	CAI	7,97	7,98	7,67	7,87	7,75	7,89	7,93	7,60	7,95	8,04	7,78	7,85	7,72	7,94	7,70	7,68	7,66	7,64	7,62	7,66	7,57	7,55	29
	CAA	24,25	24,20	25,37	24,90	25,51	24,80	24,61	26,23	24,52	24,12	25,37	24,92	25,29	24,56	25,74	26,02	26,00	26,01	25,60	25,92	26,36	26,45	28
Ile	UAI	33,59	33,56	33,56	33,52	33,55	33,56	33,56	33,56	33,56	33,56	33,53	33,56	33,56	33,55	33,56	33,56	33,56	33,53	33,54	33,56	33,55	33,56	91
	UAG	24,61	24,59	24,59	24,56	24,55	24,65	24,65	24,59	24,59	24,59	24,56	24,55	24,55	24,48	24,59	24,59	24,55	24,56	24,66	24,59	24,52	24,59	100
	UAA	27,60	27,57	27,57	27,53	27,60	27,50	27,50	27,57	27,57	27,57	27,55	27,61	27,61	27,68	27,57	27,57	27,61	27,55	27,44	27,57	27,64	27,57	33
	UAU	14,20	14,28	14,28	14,38	14,30	14,28	14,28	14,28	14,28	14,28	14,37	14,28	14,28	14,28	14,28	14,28	14,28	14,37	14,37	14,28	14,28	14,28	33
Ser	AGA	12,87	12,82	12,75	12,80	12,87	12,78	12,78	12,76	12,36	12,88	12,23	12,87	12,87	12,87	12,85	12,80	12,75	12,80	12,73	10,77	12,18	12,83	38
	AGG	11,47	11,44	11,37	11,41	11,47	11,40	11,40	11,23	10,87	11,49	10,92	11,47	11,47	11,47	11,46	11,41	11,37	11,40	11,35	9,60	10,86	11,46	100
	AGI	15,66	15,61	15,52	15,58	15,66	15,56	15,56	15,43	14,93	15,68	14,90	15,66	15,66	15,66	15,64	15,58	15,52	15,57	15,50	13,11	14,83	15,63	94
	AGU	33,33	33,46	33,95	33,68	33,33	33,77	33,77	33,92	36,04	33,37	36,51	33,33	33,33	33,33	33,29	33,48	33,95	33,81	33,93	44,19	36,87	33,37	77
	UCA	10,29	10,29	10,20	10,24	10,29	10,23	10,23	10,21	9,84	10,26	9,82	10,29	10,29	10,24	10,28	10,19	10,20	10,15	10,18	8,57	9,75	10,31	27
	UCG	6,08	6,08	6,02	6,05	6,08	6,04	6,04	6,03	5,88	6,06	5,80	6,08	6,08	6,13	6,15	6,09	6,02	6,07	6,02	5,13	5,76	6,09	97
	UCI	10,29	10,29	10,20	10,24	10,29	10,23	10,23	10,21	9,89	10,26	9,82	10,29	10,29	10,30	10,34	10,24	10,20	10,20	10,18	8,62	9,75	10,31	86
	AGC	0,00	0,00	0,00	0,00	0,00	0,00	0,00	0,20	0,19	0,00	0,00	0,00	0,00	0,00	0,00	0,20	0,00	0,00	0,10	0,00	0,00	0,00	23
Leu	AAC	27,29	27,25	27,45	27,32	27,40	27,36	27,34	27,42	26,47	27,29	28,10	27,31	27,29	27,29	27,19	27,26	27,51	27,32	27,41	28,50	28,12	27,29	26
	AAU	40,72	40,73	40,62	40,51	40,40	40,73	41,02	41,23	39,81	40,72	39,37	40,75	40,72	40,80	41,35	42,73	42,41	42,03	42,57	39,18	38,86	40,71	24
	AAI	0,00	0,00	0,00	0,00	0,00	0,00	0,00	0,00	0,00	0,00	0,00	0,00	0,00	0,00	0,00	0,00	0,00	0,00	0,00	0,00	0,00	0,00	0
	GAA	6,18	6,15	6,21	6,20	6,22	6,18	6,20	6,21	6,54	6,18	6,36	6,15	6,18	6,18	6,18	6,20	6,23	6,24	6,22	6,48	6,39	4,76	13
	GAG	3,63	3,62	3,65	3,64	3,66	3,63	3,65	3,65	3,85	3,63	3,74	3,62	3,63	3,63	3,64	3,64	3,66	3,67	3,66	3,81	3,76	4,94	44
	GAI	6,18	6,15	6,21	6,20	6,22	6,18	6,20	6,21	6,54	6,18	6,36	6,15	6,18	6,18	6,18	6,20	6,23	6,24	6,22	6,48	6,39	6,30	39
	GAC	6,06	6,13	6,10	6,08	6,07	6,09	6,09	6,09	6,42	6,06	6,31	6,07	6,06	6,06	6,13	6,08	6,05	6,04	6,10	6,33	6,40	6,06	100
	GAU	9,94	9,96	9,75	10,05	10,03	9,82	9,49	9,18	10,36	9,94	9,76	9,94	9,94	9,86	9,32	7,88	7,90	8,44	7,83	9,21	10,07	9,94	52
Gly	CCU	30,30	30,30	31,70	30,30	30,20	30,39	30,84	30,30	30,73	30,30	33,11	30,30	30,30	30,30	30,30	30,39	30,30	30,43	30,23	33,86	34,55	35,28	100
	CCI	25,06	25,05	24,52	25,12	25,23	25,08	24,86	25,26	25,30	25,07	25,22	25,06	25,06	25,12	25,23	25,21	25,21	25,19	25,33	25,26	25,23	25,29	71
	CCA	22,00	22,06	20,20	21,29	20,43	21,29	21,34	19,63	19,19	21,84	20,07	22,00	22,00	21,34	19,96	19,69	20,24	19,77	20,29	19,16	19,30	19,30	20
	CCG	17,23	17,18	17,92	17,89	18,72	17,82	17,48	19,40	19,80	17,38	18,99	17,23	17,23	17,84	19,10	19,29	18,84	19,18	18,96	19,77	19,62	19,70	82
	CCC	5,41	5,41	5,66	5,41	5,42	5,42	5,48	5,41	4,97	5,41	2,60	5,41	5,41	5,41	5,41	5,42	5,41	5,43	5,19	1,94	1,30	0,43	62
Asn	UUA	32,39	32,39	32,48	32,48	32,39	32,41	32,44	32,39	32,39	32,39	32,39	32,22	32,30	32,30	32,48	32,31	32,39	32,43	32,39	32,38	32,38	32,39	36
	UUG	30,20	30,20	30,13	30,13	30,20	30,19	30,16	30,20	30,20	30,20	30,20	30,35	30,28	30,28	30,13	30,27	30,20	30,17	30,20	30,21	30,21	30,20	100
	UUI	37,41	37,41	37,40	37,40	37,41	37,41	37,40	37,41	37,41	37,41	37,41	37,43	37,42	37,42	37,40	37,42	37,41	37,40	37,41	37,41	37,41	37,41	89
Cys	ACA	30,88	31,03	30,88	30,88	30,88	30,88	30,88	30,88	30,88	31,00	30,91	30,88	30,88	30,88	30,88	30,88	30,88	31,00	30,88	30,76	30,88	30,88	36
	ACG	29,86	29,72	29,86	29,86	29,86	29,86	29,86	29,86	29,86	29,75	29,83	29,86	29,86	29,86	29,86	29,86	29,86	29,75	29,86	29,97	29,86	29,86	100
	ACI	39,26	39,25	39,26	39,26	39,26	39,26	39,26	39,26	39,26	39,25	39,26	39,26	39,26	39,26	39,26	39,26	39,26	39,25	39,26	39,27	39,26	39,26	93
Thr	UGA	8,17	8,12	8,13	8,12	8,16	8,17	8,12	8,12	8,06	8,13	8,24	8,12	8,12	8,17	8,12	8,13	8,17	8,11	8,12	8,12	8,12	8,21	30
	UGG	16,96	17,02	17,03	17,02	16,86	16,96	17,02	17,02	16,98	17,12	16,94	17,02	17,02	16,96	17,02	16,96	16,96	16,99	17,02	17,02	17,02	16,97	100
	UGI	25,80	25,76	25,71	25,76	25,83	25,80	25,76	25,76	25,75	25,77	25,83	25,76	25,76	25,80	25,76	25,78	25,80	25,77	25,76	25,76	25,76	25,82	67
	UGU	43,75	43,78	43,70	43,78	43,84	43,75	43,78	43,78	43,89	43,77	43,67	43,78	43,78	43,75	43,78	43,81	43,75	43,83	43,78	43,78	43,78	43,69	99
	UGC	5,31	5,32	5,43	5,32	5,31	5,31	5,32	5,32	5,31	5,21	5,32	5,32	5,32	5,31	5,32	5,32	5,31	5,31	5,32	5,32	5,32	5,31	31

**C) Relative anticodon usage**		0.0 µg/ml AMD											0.05 µg/ml AMD									0.2 µg/ml AMD		
		P4	P5	P20	P36	P38	P41	P44	P65	P85	P103	P21/R	P4	P5	P20	P36	P38	P41	P44	P65	P85	P20	P38	Cell
Pro	GGA	13,63	13,63	13,76	13,81	13,95	13,72	13,58	15,17	14,17	13,66	14,76	13,66	13,63	13,72	13,80	14,13	14,58	14,58	14,58	15,33	15,72	15,90	37
	GGG	14,85	14,85	14,73	14,69	14,56	14,77	14,83	13,44	14,35	14,86	13,81	14,86	14,85	14,77	14,40	14,39	13,98	13,98	13,98	13,26	12,94	12,77	94
	GGI	18,56	18,56	18,55	18,55	18,54	18,56	18,52	18,43	18,52	18,59	18,47	18,59	18,56	18,56	18,34	18,52	18,48	18,48	18,48	18,39	18,39	18,37	89
	GGU	52,95	52,95	52,95	52,95	52,96	52,95	53,07	52,96	52,96	52,89	52,96	52,89	52,95	52,95	53,46	52,96	52,96	52,96	52,96	53,02	52,96	52,96	100
	GGC	0,00	0,00	0,00	0,00	0,00	0,00	0,00	0,00	0,00	0,00	0,00	0,00	0,00	0,00	0,00	0,00	0,00	0,00	0,00	0,00	0,00	0,00	17
Lys	UUU	74,99	74,99	74,99	74,99	74,99	74,96	74,99	74,99	74,99	74,99	75,12	74,99	74,99	75,12	74,99	74,99	74,99	74,90	74,80	75,02	74,93	74,99	64
	UUC	25,00	25,00	25,00	25,00	25,00	25,03	25,00	25,00	25,00	25,00	24,88	25,00	25,00	24,88	25,00	25,00	25,00	25,09	25,19	24,97	25,06	25,00	100
	UUI	0,00	0,00	0,00	0,00	0,00	0,00	0,00	0,00	0,00	0,00	0,00	0,00	0,00	0,00	0,00	0,00	0,00	0,00	0,00	0,00	0,00	0,00	0
His	GUA	30,88	30,76	31,03	30,88	30,88	30,88	30,88	30,94	30,88	30,85	30,88	30,88	30,88	30,88	30,76	30,88	30,82	30,51	30,88	30,88	30,88	30,88	34
	GUG	29,86	29,97	29,72	29,86	29,86	29,86	29,86	29,80	29,86	29,89	29,86	29,86	29,86	29,86	29,97	29,86	29,92	30,20	29,86	29,86	29,86	29,86	100
	GUI	39,26	39,27	39,25	39,26	39,26	39,26	39,26	39,25	39,26	39,26	39,26	39,26	39,26	39,26	39,27	39,26	39,27	39,29	39,26	39,26	39,26	39,26	92
Phe	AAA	28,90	28,90	28,95	28,95	28,79	29,15	29,14	28,95	28,95	28,95	28,95	28,90	28,95	29,00	28,95	28,95	28,95	28,76	28,97	28,95	28,90	28,95	36
	AAG	31,67	31,67	31,63	31,63	31,77	31,44	31,45	31,63	31,63	31,63	31,63	31,67	31,63	31,58	31,63	31,63	31,63	31,80	31,60	31,63	31,67	31,63	100
	AAI	39,43	39,43	39,43	39,43	39,44	39,41	39,41	39,43	39,43	39,43	39,43	39,43	39,43	39,42	39,43	39,43	39,43	39,44	39,42	39,43	39,43	39,43	93
Tyr	AUA	33,78	33,87	33,78	33,78	33,78	33,78	33,87	33,75	33,78	33,59	33,78	33,78	33,78	33,78	33,87	33,78	33,80	33,86	33,58	33,78	33,78	33,78	34,8
	AUG	27,21	27,12	27,21	27,21	27,21	27,21	27,12	27,23	27,21	27,38	27,21	27,21	27,21	27,21	27,12	27,21	27,19	27,14	27,39	27,21	27,21	27,21	100
	AUI	39,01	39,00	39,01	39,01	39,01	39,01	39,00	39,01	39,01	39,03	39,01	39,01	39,01	39,01	39,00	39,01	39,01	39,01	39,03	39,01	39,01	39,01	92,1
Asp	CUA	38,60	38,60	38,56	38,56	38,60	38,60	38,60	38,60	38,60	38,32	38,56	38,60	38,60	38,60	38,56	38,57	38,60	38,53	38,56	38,60	38,60	38,60	38,5
	CUG	22,80	22,80	22,83	22,83	22,80	22,80	22,80	22,80	22,80	23,06	22,83	22,80	22,80	22,80	22,83	22,83	22,80	22,86	22,83	22,80	22,80	22,80	100
	CUI	38,60	38,60	38,60	38,60	38,60	38,60	38,60	38,60	38,60	38,62	38,60	38,60	38,60	38,60	38,60	38,60	38,60	38,61	38,60	38,60	38,60	38,60	94,2
Glu	CUU	73,21	73,21	73,21	73,21	73,21	73,25	73,21	73,21	73,21	73,21	73,21	73,10	73,32	73,21	73,21	73,24	73,14	73,24	73,17	73,21	73,21	73,21	100
	CUI	0,00	0,00	0,00	0,00	0,00	0,00	0,00	0,00	0,00	0,00	0,00	0,00	0,00	0,00	0,00	0,00	0,00	0,00	0,00	0,00	0,00	0,00	0
	CUC	26,78	26,78	26,78	26,78	26,78	26,75	26,78	26,78	26,78	26,78	26,78	26,89	26,68	26,78	26,78	26,75	26,85	26,75	26,82	26,78	26,78	26,78	84
Gln	GUU	36,83	36,83	36,83	36,83	37,08	36,83	36,83	36,83	36,83	36,83	36,83	36,83	36,83	36,83	36,87	36,83	37,08	36,83	36,83	37,34	36,83	36,83	75
	GUI	0,00	0,00	0,00	0,00	0,00	0,00	0,00	0,00	0,00	0,00	0,00	0,00	0,00	0,00	0,00	0,00	0,00	0,00	0,00	0,00	0,00	0,00	0
	GUC	63,17	63,17	63,17	63,17	62,91	63,17	63,17	63,17	63,17	63,17	63,17	63,17	63,17	63,17	63,12	63,17	62,91	63,17	63,17	62,66	63,17	63,17	100
Ala	CGA	27,89	27,90	27,66	27,77	27,53	27,74	27,88	27,37	27,95	27,90	27,60	27,70	27,66	27,41	27,48	27,42	27,44	27,40	27,45	27,74	27,33	27,31	34
	CGG	27,10	27,10	27,11	27,14	27,09	27,11	27,03	27,13	27,20	27,10	27,12	27,11	27,11	27,13	27,13	27,13	27,13	27,24	27,20	27,33	27,14	27,14	100
	CGI	32,95	32,95	32,85	32,92	32,77	32,88	32,89	32,72	33,05	32,95	32,82	32,86	32,85	32,74	32,77	32,74	32,76	32,81	32,81	33,04	32,71	32,70	88
	CGU	12,06	12,05	12,38	12,17	12,62	12,26	12,20	12,77	11,80	12,05	12,46	12,23	12,38	12,72	12,62	12,71	12,67	12,55	12,54	11,89	12,83	12,86	70
	CGC	0,00	0,00	0,00	0,00	0,00	0,00	0,00	0,00	0,00	0,00	0,00	0,09	0,00	0,00	0,00	0,00	0,00	0,00	0,00	0,00	0,00	0,00	25
Arg	UCU	90,92	90,72	90,90	90,90	90,90	90,90	90,90	90,90	90,90	90,92	90,90	90,90	90,90	90,90	90,90	90,90	90,92	90,95	90,90	90,72	90,88	90,90	100
	UCI	0,00	0,00	0.00	0.00	0.00	0.00	0.00	0.00	0.00	0.00	0,00	0,00	0,00	0,00	0,00	0,00	0,00	0,00	0,00	0,00	0,00	0,00	0
	UCC	0,00	0.00	0,00	0,00	0,00	0,00	0,00	0,00	0,00	0,00	0,00	0,00	0,00	0,00	0,00	0,00	0,00	0,00	0,00	0,00	0,00	0,00	50
	GCG	0,00	0,00	0,00	0,00	0,00	0,00	0,00	0,00	0,00	0,00	0,00	0,00	0,00	0,00	0,00	0,00	0,00	0,00	0,00	0,00	0,00	0,00	54
	GCI	0,00	0,00	0,00	0,00	0,00	0,00	0,00	0,00	0,00	0,00	0,00	0,00	0,00	0,00	0,00	0,00	0,00	0,00	0,00	0,00	0,00	0,00	47
	GCA	0,00	0,00	0,00	0,00	0,00	0,00	0,00	0,00	0,00	0,00	0,00	0,00	0,00	0,00	0,00	0,00	0,00	0,00	0,00	0,00	0,00	0,00	13
	GCU	9,07	9,27	8,95	9,09	9,09	9,09	9,09	9,09	9,09	9,07	9,09	9,09	9,09	9,09	9,09	9,09	8,94	9,04	9,09	9,27	9,11	9,09	57
	GCC	0,00	0,00	0,14	0,00	0,00	0,00	0,00	0,00	0,00	0,00	0,00	0,00	0,00	0,00	0,00	0,00	0,14	0,00	0,00	0,00	0,00	0,00	56

**D) Anticodon variation**		0.0 µg/ml AMD											0.05 µg/ml AMD									0.2 µg/ml AMD		
		P4	P5	P20	P36	P38	P41	P44	P65	P85	P103	P21/R	P4	P5	P20	P36	P38	P41	P44	P65	P85	P20	P38	Cell
Val	CAU	0,00	0,13	-2,49	-1,88	-3,45	-1,63	-1,14	-5,32	-0,89	0,13	-3,09	-1,44	-2,49	-1,01	-4,04	-4,97	-4,74	-4,74	-2,78	-4,51	-5,66	-5,89	74
	CAC	0,00	0,13	-2,49	-1,88	-3,45	-1,63	-1,14	-5,32	-0,89	0,13	-3,09	-2,12	-2,49	-1,01	-4,04	-4,97	-4,88	-4,74	-3,32	-4,51	-5,66	-5,89	100
	CAG	0,00	-0,06	0,95	0,97	1,70	0,86	0,63	2,56	0,52	0,05	1,53	0,90	1,05	0,57	1,97	2,50	2,38	2,30	1,23	2,19	2,72	2,83	57
	CAI	0,00	0,13	-3,80	-1,22	-2,80	-0,97	-0,47	-4,68	-0,22	0,80	-2,44	-1,47	-3,14	-0,35	-3,40	-3,70	-3,85	-4,10	-4,35	-3,87	-5,03	-5,26	29
	CAA	0,00	-0,20	4,61	2,67	5,18	2,28	1,48	8,16	1,08	-0,54	4,61	2,74	4,29	1,28	6,12	7,30	7,21	7,24	5,54	6,87	8,71	9,07	28
Ile	UAI	0,00	-0,10	-0,10	-0,20	-0,12	-0,08	-0,08	-0,10	-0,10	-0,10	-0,19	-0,10	-0,10	-0,12	-0,10	-0,10	-0,10	-0,19	-0,16	-0,10	-0,11	-0,10	91
	UAG	0,00	-0,10	-0,10	-0,18	-0,23	0,18	0,18	-0,10	-0,10	-0,10	-0,19	-0,23	-0,23	-0,51	-0,10	-0,10	-0,23	-0,19	0,22	-0,10	-0,37	-0,10	100
	UAA	0,00	-0,10	-0,10	-0,23	0,00	-0,36	-0,36	-0,10	-0,10	-0,10	-0,19	0,04	0,04	0,30	-0,10	-0,10	0,04	-0,19	-0,59	-0,10	0,17	-0,10	33
	UAU	0,00	0,58	0,58	1,25	0,67	0,58	0,58	0,58	0,58	0,58	1,15	0,58	0,58	0,58	0,58	0,58	0,58	1,15	1,15	0,58	0,58	0,58	33
Ser	AGA	0,00	-0,40	-0,92	-0,53	0,00	-0,66	-0,66	-0,79	-3,97	0,13	-4,96	0,00	0,00	0,00	-0,13	-0,53	-0,92	-0,53	-1,06	-16,29	-5,30	-0,27	38
	AGG	0,00	-0,26	-0,92	-0,53	0,00	-0,66	-0,66	-2,13	-5,27	0,13	-4,83	0,00	0,00	0,00	-0,13	-0,53	-0,92	-0,66	-1,06	-16,29	-5,30	-0,13	100
	AGI	0,00	-0,33	-0,92	-0,53	0,00	-0,66	-0,66	-1,50	-4,65	0,13	-4,89	0,00	0,00	0,00	-0,13	-0,53	-0,92	-0,60	-1,05	-16,28	-5,30	-0,20	94
	AGU	0,00	0,40	1,85	1,06	0,00	1,32	1,32	1,78	8,12	0,13	9,54	0,00	0,00	0,00	-0,13	0,46	1,85	1,45	1,81	32,59	10,61	0,13	77
	UCA	0,00	0,00	-0,92	-0,53	0,00	-0,66	-0,66	-0,79	-4,45	-0,37	-4,58	0,00	0,00	-0,50	-0,13	-1,03	-0,92	-1,42	-1,06	-16,71	-5,30	0,13	27
	UCG	0,00	0,00	-0,92	-0,53	0,00	-0,66	-0,66	-0,79	-3,23	-0,37	-4,58	0,00	0,00	0,77	1,14	0,24	-0,92	-0,16	-1,06	-15,65	-5,30	0,13	97
	UCI	0,00	0,00	-0,92	-0,53	0,00	-0,66	-0,66	-0,79	-3,93	-0,37	-4,58	0,00	0,00	0,04	0,41	-0,49	-0,92	-0,88	-1,06	-16,26	-5,30	0,13	86
	AGC																							23
Leu	AAC	0,00	-0,14	0,56	0,10	0,38	0,26	0,18	0,48	-3,01	0,00	2,97	0,08	0,00	0,00	-0,36	-0,12	0,81	0,12	0,42	4,43	3,02	0,00	26
	AAU	0,00	0,03	-0,23	-0,52	-0,78	0,04	0,75	1,27	-2,23	0,00	-3,31	0,08	0,00	0,20	1,56	4,96	4,17	3,23	4,55	-3,78	-4,55	0,00	24
	AAI																							0
	GAA	0,00	-0,42	0,56	0,32	0,66	-0,02	0,40	0,48	5,93	0,00	2,97	-0,42	0,00	0,00	0,08	0,32	0,81	1,07	0,64	4,95	3,48	-23,00	13
	GAG	0,00	-0,42	0,56	0,32	0,66	-0,02	0,40	0,48	5,93	0,00	2,97	-0,42	0,00	0,00	0,08	0,32	0,81	1,07	0,64	4,95	3,48	35,86	44
	GAI	0,00	-0,42	0,56	0,32	0,66	-0,02	0,40	0,48	5,93	0,00	2,97	-0,42	0,00	0,00	0,08	0,32	0,80	1,06	0,64	4,95	3,48	1,96	39
	GAC	0,00	1,08	0,56	0,32	0,16	0,48	0,40	0,48	5,93	0,00	4,00	0,08	0,00	0,00	1,08	0,32	-0,20	-0,44	0,64	4,43	5,55	0,00	100
	GAU	0,00	0,28	-1,87	1,13	0,97	-1,14	-4,45	-7,61	4,23	0,00	-1,81	0,08	0,00	-0,81	-6,17	-20,68	-20,49	-15,02	-21,23	-7,34	1,38	0,00	52
Gly	CCU	0,00	0,00	4,63	0,00	-0,33	0,29	1,78	0,00	1,43	0,00	9,29	0,00	0,00	0,00	0,00	0,29	0,00	0,43	-0,23	11,74	14,05	16,43	100
	CCI	0,00	-0,02	-2,15	0,24	0,69	0,07	-0,79	0,81	0,96	0,06	0,66	0,00	0,00	0,23	0,70	0,62	0,60	0,50	1,06	0,81	0,68	0,92	71
	CCA	0,00	0,25	-8,22	-3,26	-7,14	-3,23	-3,01	-10,78	-12,78	-0,75	-8,78	0,00	0,00	-3,01	-9,27	-10,52	-8,02	-10,14	-7,77	-12,91	-12,29	-12,29	20
	CCG	0,00	-0,29	4,01	3,81	8,64	3,43	1,46	12,59	14,93	0,88	10,24	0,00	0,00	3,51	10,83	11,94	9,37	11,33	10,04	14,76	13,85	14,33	82
	CCC	0,00	0,00	4,63	0,00	0,14	0,29	1,30	0,00	-8,00	0,00	-5,60	0,00	0,00	0,00	0,00	0,29	0,00	0,43	-4,00	-6,40	-7,60	-6,60	62
Asn	UUA	0,00	0,00	0,27	0,27	0,00	0,07	0,17	0,00	0,00	0,00	0,00	-0,51	-0,27	-0,27	0,27	-0,24	0,00	0,14	0,00	-0,03	-0,03	0,00	36
	UUG	0,00	0,00	-0,25	-0,25	0,00	-0,06	-0,16	0,00	0,00	0,00	0,00	0,47	0,25	0,25	-0,25	0,22	0,00	-0,13	0,00	0,03	0,03	0,00	100
	UUI	0,00	0,00	-0,03	-0,03	0,00	-0,01	-0,02	0,00	0,00	0,00	0,00	0,06	0,03	0,03	-0,03	0,03	0,00	-0,02	0,00	0,00	0,00	0,00	89
Cys	ACA	0,00	0,50	0,00	0,00	0,00	0,00	0,00	0,00	0,00	0,40	0,10	0,00	0,00	0,00	0,00	0,00	0,00	0,40	0,00	-0,40	0,00	0,00	36
	ACG	0,00	-0,47	0,00	0,00	0,00	0,00	0,00	0,00	0,00	-0,38	-0,09	0,00	0,00	0,00	0,00	0,00	0,00	-0,38	0,00	0,38	0,00	0,00	100
	ACI	0,00	-0,03	0,00	0,00	0,00	0,00	0,00	0,00	0,00	-0,03	-0,01	0,00	0,00	0,00	0,00	0,00	0,00	-0,03	0,00	0,03	0,00	0,00	93
Thr	UGA	0,00	-0,60	-0,55	-0,60	-0,12	0,00	-0,60	-0,60	-1,32	-0,55	0,80	-0,60	-0,60	0,00	-0,60	-0,52	0,00	-0,80	-0,60	-0,60	-0,60	0,52	30
	UGG	0,00	0,35	0,41	0,35	-0,60	0,00	0,35	0,35	0,10	0,89	-0,15	0,35	0,35	0,00	0,35	-0,05	0,00	0,15	0,35	0,35	0,35	0,05	100
	UGI	0,00	-0,15	-0,32	-0,15	0,11	0,00	-0,15	-0,15	-0,19	-0,09	0,12	-0,15	-0,15	0,00	-0,15	-0,07	0,00	-0,12	-0,15	-0,15	-0,15	0,07	67
	UGU	0,00	0,06	-0,13	0,06	0,20	0,00	0,06	0,06	0,32	0,04	-0,18	0,06	0,06	0,00	0,06	0,14	0,00	0,18	0,06	0,06	0,06	-0,14	99
	UGC	0,00	0,06	2,12	0,06	-0,12	0,00	0,06	0,06	0,00	-1,88	0,14	0,06	0,06	0,00	0,06	0,14	0,00	-0,14	0,06	0,06	0,06	-0,14	31

**D) Anticodon variation**		0.0 µg/ml AMD											0.05 µg/ml AMD									0.2 µg/ml AMD		
		P4	P5	P20	P36	P38	P41	P44	P65	P85	P103	P21/R	P4	P5	P20	P36	P38	P41	P44	P65	P85	P20	P38	Cell
Pro	GGA	0,00	0,00	1,00	1,33	2,33	0,67	-0,33	11,34	4,00	0,22	8,34	0,22	0,00	0,67	1,29	3,67	7,00	7,00	7,00	12,47	15,34	16,67	37
	GGG	0,00	0,00	-0,84	-1,12	-1,96	-0,56	-0,18	-9,53	-3,36	0,06	-7,01	0,06	0,00	-0,56	-3,05	-3,08	-5,89	-5,89	-5,89	-10,73	-12,90	-14,02	94
	GGI	0,00	0,00	-0,06	-0,08	-0,15	-0,04	-0,24	-0,71	-0,25	0,13	-0,52	0,13	0,00	-0,04	-1,22	-0,23	-0,44	-0,44	-0,44	-0,92	-0,96	-1,04	89
	GGU	0,00	0,00	0,00	0,00	0,00	0,00	0,22	0,00	0,00	-0,12	0,00	-0,12	0,00	0,00	0,95	0,00	0,00	0,00	0,00	0,12	0,01	0,01	100
	GGC																							17
Lys	UUU	0,00	0,00	0,00	0,00	0,00	-0,04	0,00	0,00	0,00	0,00	0,17	0,00	0,00	0,17	0,00	0,00	0,00	-0,13	-0,25	0,04	-0,08	0,00	64
	UUC	0,00	0,00	0,00	0,00	0,00	0,13	0,00	0,00	0,00	0,00	-0,50	0,00	0,00	-0,50	0,00	0,00	0,00	0,38	0,75	-0,12	0,25	0,00	100
	UUI																							0
His	GUA	0,00	-0,40	0,50	0,00	0,00	0,00	0,00	0,20	0,00	-0,10	0,00	0,00	0,00	0,00	-0,40	0,00	-0,20	-1,21	0,00	0,00	0,00	0,00	34
	GUG	0,00	0,38	-0,47	0,00	0,00	0,00	0,00	-0,19	0,00	0,09	0,00	0,00	0,00	0,00	0,38	0,00	0,19	1,14	0,00	0,00	0,00	0,00	100
	GUI	0,00	0,03	-0,03	0,00	0,00	0,00	0,00	-0,01	0,00	0,01	0,00	0,00	0,00	0,00	0,03	0,00	0,01	0,08	0,00	0,00	0,00	0,00	92
Phe	AAA	0,00	0,00	0,17	0,17	-0,37	0,88	0,83	0,17	0,17	0,17	0,17	0,00	0,17	0,33	0,17	0,17	0,17	-0,50	0,25	0,17	0,00	0,17	36
	AAG	0,00	0,00	-0,14	-0,14	0,31	-0,73	-0,70	-0,14	-0,14	-0,14	-0,14	0,00	-0,14	-0,28	-0,14	-0,14	-0,14	0,42	-0,21	-0,14	0,00	-0,14	100
	AAI	0,00	0,00	-0,01	-0,01	0,02	-0,05	-0,05	-0,01	-0,01	-0,01	-0,01	0,00	-0,01	-0,02	-0,01	-0,01	-0,01	0,03	-0,02	-0,01	0,00	-0,01	93
Tyr	AUA	0,00	0,29	0,00	0,00	0,00	0,00	0,29	-0,07	0,00	-0,53	0,00	0,00	0,00	0,00	0,29	0,00	0,07	0,25	-0,57	0,00	0,00	0,00	34,8
	AUG	0,00	-0,32	0,00	0,00	0,00	0,00	-0,32	0,08	0,00	0,61	0,00	0,00	0,00	0,00	-0,32	0,00	-0,08	-0,28	0,65	0,00	0,00	0,00	100,0
	AUI	0,00	-0,02	0,00	0,00	0,00	0,00	-0,02	0,01	0,00	0,04	0,00	0,00	0,00	0,00	-0,02	0,00	-0,01	-0,02	0,04	0,00	0,00	0,00	92,1
Asp	CUA	0,00	0,00	-0,09	-0,09	0,00	0,00	0,00	0,00	0,00	-0,72	-0,09	0,00	0,00	0,00	-0,09	-0,09	0,00	-0,18	-0,09	0,00	0,00	0,00	38,5
	CUG	0,00	0,00	0,14	0,14	0,00	0,00	0,00	0,00	0,00	1,12	0,14	0,00	0,00	0,00	0,14	0,14	0,00	0,28	0,14	0,00	0,00	0,00	100,0
	CUI	0,00	0,00	0,01	0,01	0,00	0,00	0,00	0,00	0,00	0,06	0,01	0,00	0,00	0,00	0,01	0,01	0,00	0,02	0,01	0,00	0,00	0,00	94,2
Glu	CUU	0,00	0,00	0,00	0,00	0,00	0,05	0,00	0,00	0,00	0,00	0,00	-0,15	0,15	0,00	0,00	0,04	-0,09	0,04	-0,05	0,00	0,00	0,00	100
	CUI																							0
	CUC	0,00	0,00	0,00	0,00	0,00	-0,14	0,00	0,00	0,00	0,00	0,00	0,40	-0,40	0,00	0,00	-0,12	0,26	-0,12	0,14	0,00	0,00	0,00	84
Gln	GUU	0,00	0,00	0,00	0,00	0,69	0,00	0,00	0,00	0,00	0,00	0,00	0,00	0,00	0,00	0,11	0,00	0,69	0,00	0,00	1,37	0,00	0,00	75
	GUI																							0
	GUC	0,00	0,00	0,00	0,00	-0,40	0,00	0,00	0,00	0,00	0,00	0,00	0,00	0,00	0,00	-0,07	0,00	-0,40	0,00	0,00	-0,80	0,00	0,00	100
Ala	CGA	0,00	0,04	-0,83	-0,45	-1,29	-0,53	-0,04	-1,88	0,20	0,04	-1,04	-0,70	-0,83	-1,74	-1,48	-1,70	-1,61	-1,78	-1,59	-0,55	-2,01	-2,10	34
	CGG	0,00	0,00	0,06	0,16	-0,04	0,04	-0,26	0,14	0,38	0,00	0,07	0,05	0,06	0,13	0,11	0,12	0,12	0,54	0,38	0,85	0,15	0,15	100
	CGI	0,00	0,01	-0,30	-0,09	-0,55	-0,19	-0,17	-0,68	0,31	0,01	-0,38	-0,25	-0,30	-0,63	-0,54	-0,62	-0,59	-0,41	-0,42	0,28	-0,73	-0,77	88
	CGU	0,00	-0,13	2,60	0,91	4,58	1,67	1,14	5,90	-2,15	-0,13	3,27	1,43	2,60	5,48	4,64	5,34	5,06	4,01	3,96	-1,42	6,33	6,62	70
	CGC																							25
Arg	UCU	0,00	-0,22	-0,02	-0,02	-0,02	-0,02	-0,02	-0,02	-0,02	0,00	-0,02	-0,02	-0,02	-0,02	-0,02	-0,02	0,00	0,04	-0,02	-0,22	-0,04	-0,02	100
	UCI																							0
	UCC																							50
	GCG																							54
	GCI																							47
	GCA																							13
	GCU	0,00	2,19	-1,34	0,18	0,18	0,18	0,18	0,18	0,18	0,00	0,18	0,18	0,18	0,18	0,18	0,18	-1,52	-0,36	0,18	2,19	0,36	0,18	57
	GCC																							56

Those anticodons whose initial counts were 0 were omitted in the analysis of the anticodon usage variation.

Those anticodons whose variation was exceptional (only in a few pasages) and inconsistent were omitted in the analysis of the anticodon usage variation.
