# Supplementary figures and images for: Fine-Tuning Translation Kinetics Selection as the Driving Force of Codon Usage Bias in the Hepatitis A Virus Capsid
Source: PLoS Pathog. 2010 Mar 5;6(3):e1000797. doi: 10.1371/journal.ppat.1000797 (PMC2832697; doi:10.1371/journal.ppat.1000797)

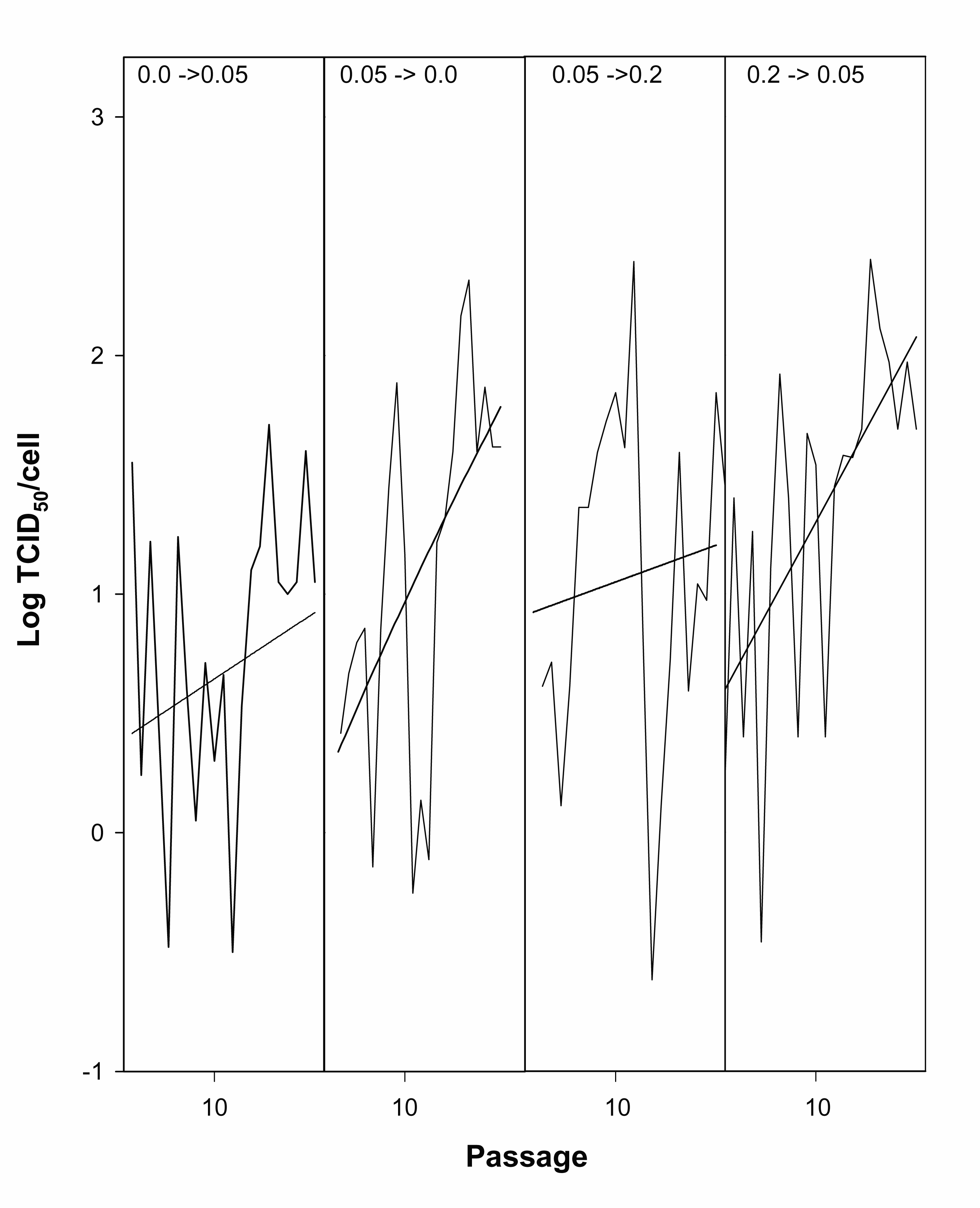

Supplement: Figure S1 — Infectious HAV titer production per cell during the first 20 adaptation passages of lineages 1 (adaptation to 0.05 µg/ml of AMD) and 3 (adaptation from 0.05 µg/ml to 0.0 µg/ml of AMD), and lineages 2 (adaptation from 0.05 µg/ml to 0.2 µg/ml of AMD) and 4 (adaptation from 0.2 µg/ml to 0.05 µg/ml of AMD). Linear regression analysis showed that the kinetics of adaptation were faster (with significant differences in the slopes, p<0.05) in the re-adaptation to the original conditions than in the first adaptation to the different AMD concentrations. (0.99 MB TIF) [file ppat.1000797.s006.tif]

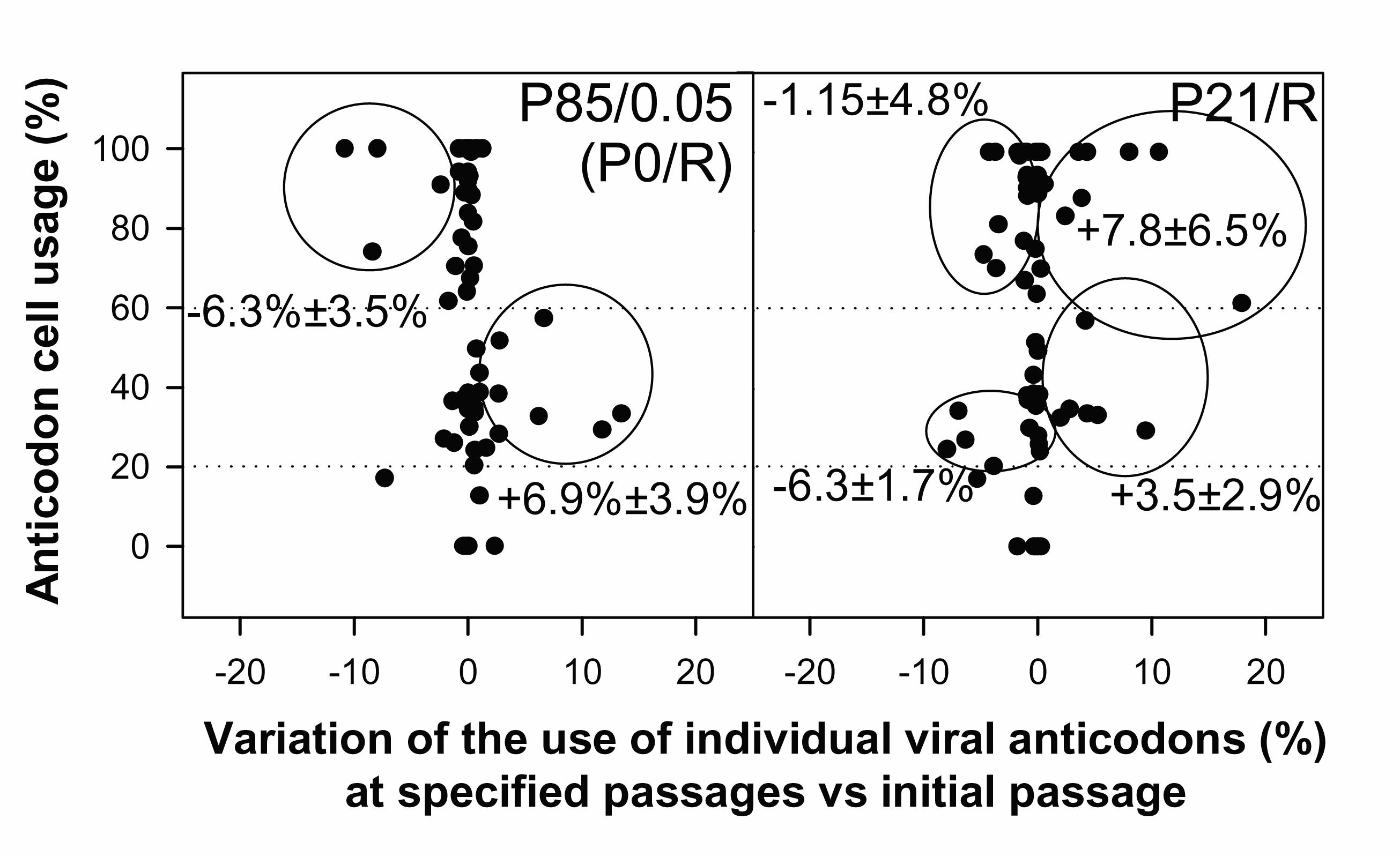

Supplement: Figure S2 — Re-adaptation from 0.05 to 0.0 µg/ml of AMD. Right panel depicts the behavior of the population re-adapting from 0.05 to 0.0 µg/ml of AMD (lineage 3). Left panel depicts the behavior of the population in 0.05 µg/ml of AMD as baseline control. The percentage of variation of each individual viral anticodon in the mutant spectra with respect to the cellular usage is shown. Mean and standard deviation of those anticodons with the highest level of variation (encircled) is shown. (0.58 MB TIF) [file ppat.1000797.s007.tif]

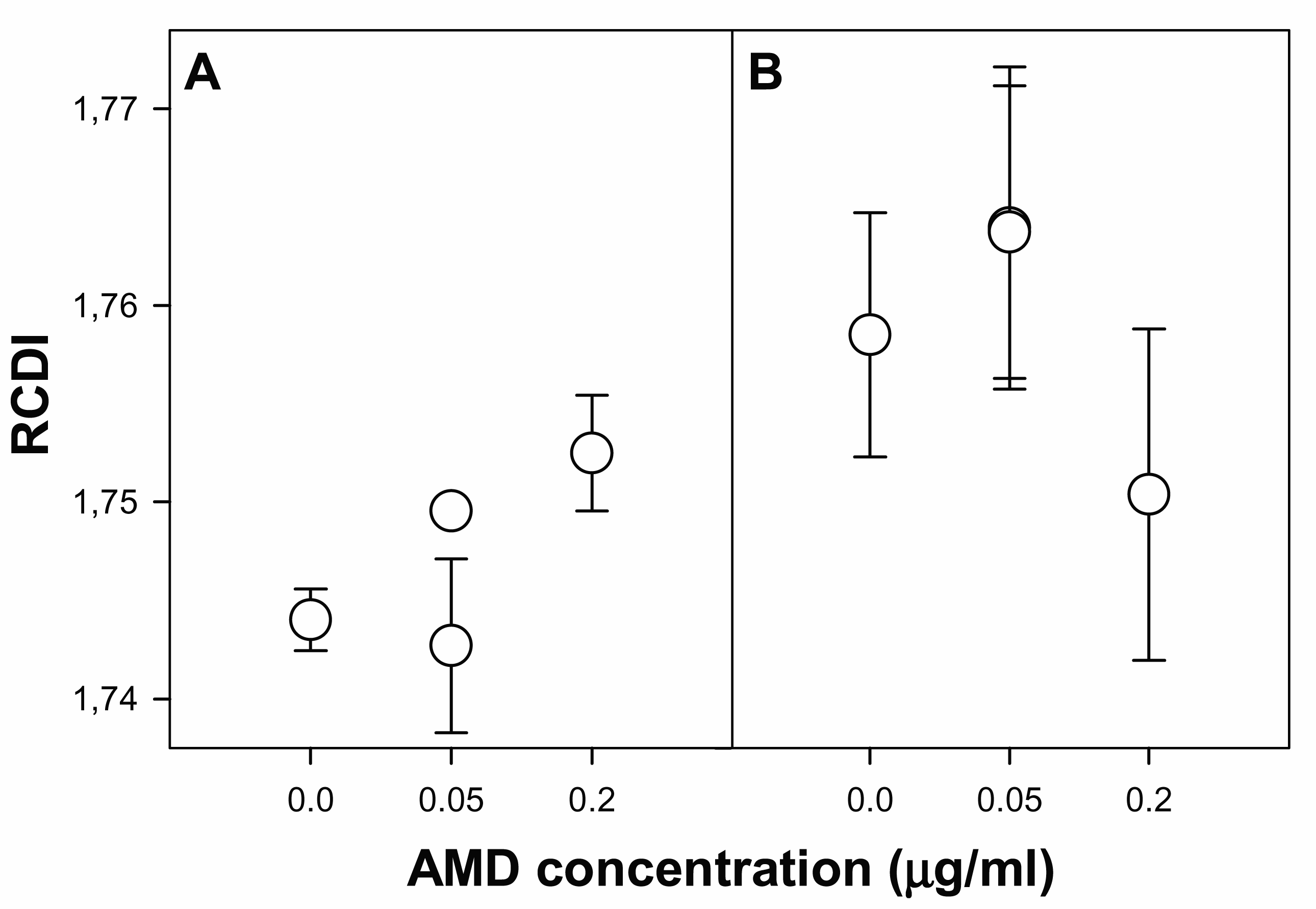

Supplement: Figure S3 — Relative codon deoptimization index (RCDI) during the process of adaptation to AMD. A. RCDI (mean±SD) in the capsid coding region. B. RCDI (mean±SD) in the polymerase coding region. During the process of adaptation to 0.05 µ g/ml of AMD no differences were detected until P44 (hence two values are given). The first value represents passages P1-P44 and the second passages P45–P103. As it can be observed, the capsid region showed a significant increase of the RCDI during the process of adaptation to increasing AMD concentrations. On the contrary, the RCDI of the polymerase coding region although showing a tendency to decrease did not significantly vary. (0.49 MB TIF) [file ppat.1000797.s008.tif]
